# *Brassica rapa* BrICE1 and BrICE2 Positively Regulate the Cold Tolerance via CBF and ROS Pathways, Balancing Growth and Defense in Transgenic Arabidopsis

**DOI:** 10.3390/plants13182625

**Published:** 2024-09-20

**Authors:** Wangze Wu, Haobo Yang, Peng Xing, Guoting Zhu, Xueyan Han, Mei Xue, Guotai Min, Haijun Ding, Guofan Wu, Zigang Liu

**Affiliations:** 1 College of Life Sciences, Northwest Normal University, Lanzhou 730070, China; xxfxpxp@163.com (P.X.); zhuguo2022212@163.com (G.Z.); 2021212777@nwnu.edu.cn (X.H.); 2023212787@nwnu.edu.cn (M.X.); 202131901405@nwnu.edu.cn (G.M.); hh7777rose@163.com (H.D.); wugf1971@163.com (G.W.); 2 Guangdong Provincial Key Laboratory of Plant Adaptation and Molecular Design, School of Life Sciences, Guangzhou University, Guangzhou 510006, China; yhb998125@163.com; 3 State Key Laboratory of Aridland Crop Science, College of Agronomy, Gansu Agricultural University, Lanzhou 730070, China; lzgworking@163.com

**Keywords:** BrICE1, BrICE2, freezing tolerance, phylogenetic tree, CBF pathway, *Brassica rapa*

## Abstract

Winter rapeseed (*Brassica rapa*) has a good chilling and freezing tolerance. inducer of CBF expression 1 (ICE1) plays a crucial role in cold signaling in plants; however, its role in *Brassica rapa* remains unclear. In this study, we identified 41 *ICE1* homologous genes from six widely cultivated Brassica species. These genes exhibited high conservation, with evolutionary complexity between diploid and allotetraploid species. Cold stress induced *ICE1* homolog expression, with differences between strongly and weakly cold-tolerant varieties. Two novel *ICE1* paralogs, *BrICE1* and *BrICE2*, were cloned from *Brassica rapa* Longyou 6. Subcellular localization assays showed that they localized to the nucleus, and low temperature did not affect their nuclear localization. The overexpression of *BrICE1* and *BrICE2* increased cold tolerance in transgenic Arabidopsis and enhanced reactive oxygen species’ (ROS) scavenging ability. Furthermore, our data demonstrate that overexpression of *BrICE1* and *BrICE2* inhibited root growth in Arabidopsis, and low temperatures could induce the degradation of BrICE1 and BrICE2 via the 26S-proteasome pathway. In summary, *ICE1* homologous genes exhibit complex evolutionary relationships in Brassica species and are involved in the C-repeat/DREB binding factor (CBF) pathway and ROS scavenging mechanism in response to cold stress; these regulating mechanisms might also be responsible for balancing the development and cold defense of *Brassica rapa*.

## 1. Introduction

Low temperatures are a major ecological and environmental factor that strongly affects plant development and geographic distribution. Low-temperature stress is categorized into chilling stress (0–15 °C) and freezing stress (<0 °C) [1,2]. Chilling and freezing stresses are two kinds of different stresses, and require different solutions [2]. Chilling damage is a direct temperature effect that mainly restricts the growth and development of a plant, including wilting, chlorosis, sterility and even death. Nevertheless, freezing damage results from cellular dehydration and membrane injury triggered by extracellular ice crystallization. Thus, sudden freezing damage is more damaging to plants than chilling stress [2]. However, plants have evolved a series of sophisticated regulatory mechanisms to adapt to low-temperature stress. Among them, the most important regulatory mechanism is termed cold acclimation (or cold hardening), in which prior exposure of plants to low but non-freezing temperatures can enhance the freezing tolerance of the plant [2]. In this process, a series of physiological and molecular changes take place in their cells, including synthesizing protective osmotic substances (soluble sugars, proline) and cold-resistance proteins (late embryogenesis abundant, LEA; antifreeze protein, AFP) [3]. These substances and cold-resistance proteins participate in osmoregulation, reactive oxygen species (ROS) scavenging and ice crystal formation [4]. In fact, cold-stress-triggered physiological and molecular changes rely in part on gene induction by transcriptional and post-transcriptional regulation. To date, the best-characterized cold-signaling pathway is the ICE1-CBF-COR regulatory cascade.

In the ICE1-CBF-COR cascade, the induction of cold-regulated (*COR*) genes is an important biological event [5]. *COR* genes encode key enzymes and cryoprotective proteins, such as soluble sugars, soluble proteins and proline, which protect plant cells against cold-induced damage [2]. Under low-temperature stress, the CBF is rapidly induced and regulates the expression of downstream *COR* genes by binding to their promoter regions [6,7,8]. CBFs are key upstream transcription regulators of *COR* genes, and their expression can be accurately controlled by upstream transcription factors. Among these factors, ICE1, a MYC-like basic helix–loop–helix (bHLH) transcription factor, is the best-characterized positive regulator of *CBF* genes identified to date [6,7,8].

Under cold stress, ICE1 directly binds to the MYC recognition motif of the CBF3 promoter, leading to the activation of *CBF3* expression [6]. In Arabidopsis, loss-of-function mutations in ICE1 lead to reduced resistance to cold stress, whereas ICE1 overexpression enhances the cold-induced upregulation of *CBFs* [6]. ICE2, a paralog of ICE1 with 61% identity, shares similar functions. Overexpression of either *ICE1* or *ICE2* enhances freezing resistance in transgenic Arabidopsis [6,9,10]. Notably, ICE1 primarily regulates *CBF3* expression in cold signaling [6], while ICE2 mainly targets *CBF1* [10]. This suggests functional redundancy with distinct downstream targets. However, other studies using loss-of-function mutants suggest ICE1 may play a more dominant role compared with ICE2 [11].

Although ICE1 is the key regulator in the ICE1–CBF–COR cold-signaling pathway, *ICE1* expression itself is not responsive to cold at the transcriptional level [6]. Its activity is controlled at the protein level by the 26S-proteasome pathway [12,13], highlighting the importance of post-translational modifications (PTMs). Emerging evidence indicates that multiple PTMs control ICE1 cell turnover and duration at low temperatures. High expression of osmotically responsive gene 1 (HOS1), a ubiquitin E3 ligase with a RING finger, directly interacts with ICE1, promoting its degradation and negatively regulating cold resistance [13]. Conversely, SAP and Miz1 domain-containing ligase 1 (SIZ1), a SUMO E3 ligase, enhances cold tolerance by stabilizing ICE1 through SUMOylation, which reduces HOS1-mediated ubiquitination [14]. Open stomata 1 (OST1), a Ser/Thr protein kinase involved in abscisic acid (ABA) signaling, can be activated by cold stress. Cold-activated OST1 phosphorylates ICE1 and enhances its stability by interfering with the interaction between HOS1 and ICE1, thereby enhancing freezing tolerance [12]. Beyond their role in cold signaling, ICE1/SCREAM (SCRM) and ICE2/SCRM2 are also involved in diverse processes, including stomatal development [15,16], flowering [17], primary seed dormancy [18], male fertility and ABA signaling [19]. These findings suggest that ICE1 is not only a central component in the ICE1–CBF–COR cold-signaling pathway but also serves as a convergence point, integrating multiple signals to regulate both cold tolerance and plant growth.

Given its crucial role, ICE1 homologs have been identified in numerous plants, including wheat (*Triticum aestivum*) [20], rice (*Oryza sativa*) [21], *Saussurea involucrata* [22], maize (*Zea mays*) [23] and tomato (*Solanum lycopersicum*) [24]. Although these ICE1 homologous genes are involved in cold stress, different species may display diverse ICE1-dependent cold-regulation mechanisms [25,26,27]. For example, in Arabidopsis, cold-activated mitogen-activated protein kinase 3 (MPK3) and MPK6 kinases phosphorylate ICE1, targeting it for degradation and negatively regulating cold responses [26,27]. However, in rice, cold-activated OsMAPK3 phosphorylates and stabilizes OsbHLH002 (a rice ICE1 homolog), leading to trehalose-6-phosphate phosphatase (OsTPP1) activation and increased OsTPP1-regulated trehalose content [25]. These contrasting findings indicate that ICE1 is evolutionarily conserved but contains functional divergence in cold signals in different species, particularly between Arabidopsis and rice. This functional differentiation of ICE1 is supported by a recent study in which *PsnICE1*, a poplar ICE1 homolog (*Populus tomentosa Carr*), was not only involved in the CBF-dependent pathway but also in ROS scavenging in response to cold stress by binding to different cis-acting elements [28].

In addition to the functional diversity of different species, conflicting results exist regarding Arabidopsis ICE1’s role in cold signaling. For example, *ice1* (a single substitution mutant of Arg-236 to His), a well-known dominant-negative mutant, exhibits reduced chilling and freezing tolerance, regardless of cold acclimation [6]. However, another study found that *ice1-2* and *ice2-2*, two T-DNA insertional mutants, did not exhibit any sensitive freezing tolerance phenotype in the absence of cold acclimation, suggesting that ICE1 and ICE2 may not be involved in the basal cold resistance of plants [11].

Over the past three decades, the biological function of ICE1 has been well understood in Arabidopsis and rice; however, as neither are winter plants, they cannot survive safely in winter. In contrast, some winter crops, such as winter rapeseed (*Brassica rapa*), an important oilseed and economic crop worldwide, can survive at extremely low temperatures (−20 °C to −32 °C) [29,30]. Theoretically, these winter *Brassica rapa* crops may have evolved more effective cold-acclimation mechanisms to respond to chilling and freezing stresses; however, the fundamental molecular mechanism remains elusive in *Brassica rapa*. Until recently, using multi-omics technology, some cold-responsive differentially expressed genes (DEGs) [29,31,32,33], microRNA [34,35] and differentially accumulated proteins (DAPs) [36,37] have been identified in *Brassica rapa*. Nevertheless, these omics studies still cannot reveal the detailed molecular mechanism that dictates *Brassica rapa*’s response to cold stress at a molecular level. Unlike in Arabidopsis and rice, to date, only a few cold-regulated genes have been identified in *Brassica napus* (*BN115*, *BnCBF17*, *BnHOS1*) [38,39,40], but not in *Brassica rapa*. Recently, several studies have started to try to explore the molecular mechanism of *Brassica rapa*’s response to cold stress. For instance, Dong et al. found that overexpression of *Brassica rapa* antifreeze protein 1 (BrAFP1) increased the cold tolerance of transgenic Arabidopsis [41]. Our previous study using transcriptome analysis and immunoblotting assays revealed that MAPK kinase and Ca^2+^-related protein kinase are important signaling molecules under low-temperature stress in *Brassica rapa* [31]. Subsequently, our study also found that *Brassica rapa* EIN3-binding f-box 1 (BrEBF1) positively regulated cold tolerance, and BrEBF1-regulated cold tolerance is associated with ROS scavenging and MAPK kinase activity [42].

Considering ICE1’s role in cold signaling, some researchers have investigated its physiological functions in *Brassica rapa*. However, unlike in Arabidopsis, the phylogenetic, evolutionary and physiological functional divergence of ICE1 paralogs in cold signals remains unknown. In this study, the ICE1 homolog genes of six widely cultivated Brassica species were identified, and the role of BrICE1 and BrICE2 of *Brassica rapa* in cold signaling were investigated. Our studies discovered that *ICE1* homologous genes exhibit complex evolutionary relationships in Brassica species; two novel BrICE1 and BrICE2 paralogs of *Brassica rapa* positively regulate its cold tolerance via a CBF pathway and ROS scavenging mechanism, which can balance the development and cold defense in transgenic Arabidopsis.

## 2. Results

### 2.1. Identification and Phylogenetic Analysis of ICE1 Homologous Genes in Brassica Species

**Figure 1:**
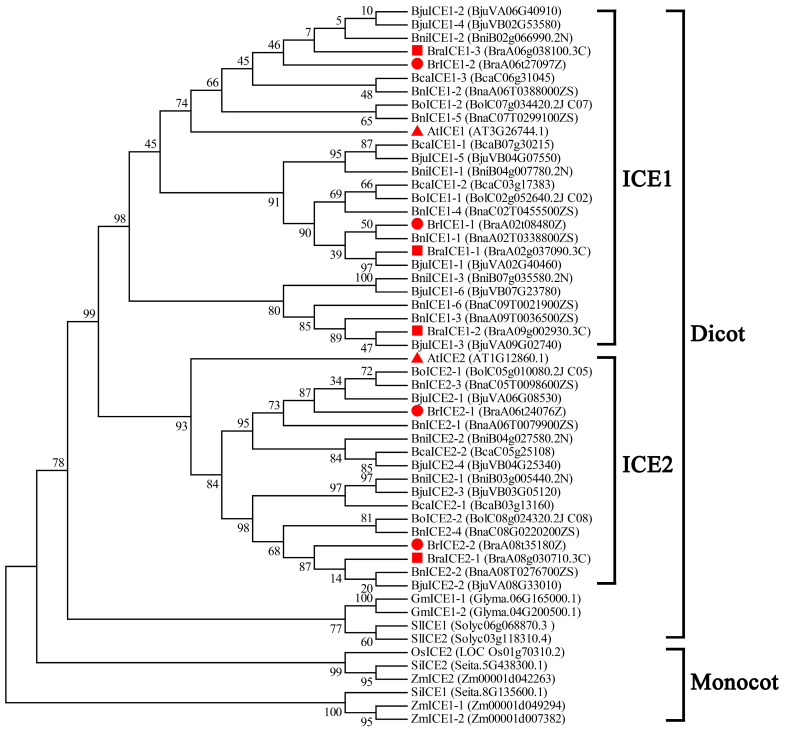
Phylogenetic analysis of *ICE1* homologous genes in Brassica species. The phylogenetic tree was constructed by neighbor-joining distance using MEGA 6.0. A total of 42 *ICE1* homologous genes were identified from *Brassica* species. Well-known *ICE1* and *ICE2* homologous genes of dicotyledon *Arabidopsis thaliana*, tomato (*Solanum lycopersicum*), soybean (*Glycine max*), monocotyledon maize (*Zea mays*), foxtail millet (*Setaria italica*) and rice (*Oryza sativa*) were used as the outgroup. *BrICE*, *BraICE*, *BoICE*, *BniICE*, *BnICE*, *BjuICE* and *BcaICE* stand for the *ICE1* homologous genes of Z1 (*B. rapa*, yellow sarson, as an oilseed crop), Chiifu-401-42 (*B. rapa*, Chinese cabbage, as a vegetable), *B. oleracea*, *B. nigra*, *B. napus*, *B. juncea* and *B. carinata*, respectively. The red-filled triangle, red-filled square and red-filled circle represent *ICE1* homologous genes of *Arabidopsis*, *B. rapa* (Chiifu-401-42, as a vegetable) and *B. rapa* (Z1, as an oilseed crop).

Brassica species belong to the Brassica genus of the Brassicaceae family, which contains a diverse range of oilseed and vegetable crops in the word [43]. Six Brassica crops, including three diploid species, *Brassica rapa* (AA), *Brassica nigra* (BB) and *Brassica oleracea* (CC), and three allopolyploid species, *Brassica napus* (AACC), *Brassica juncea* (AABB) and *Brassica carinata* (BBCC), are extensively cultivated around the world [44]. Low temperatures severely affect the yield and quality of Brassica species. Several studies have demonstrated that ICE1 of *B. rapa* is involved in cold-pathway signaling [45,46]. However, no study has reported the functional redundancy and structural evolutionary relationship between ICE1 and ICE2 in Brassica species under cold signals. To identify the homologous genes of *ICE1* in the six widely cultivated Brassica species (*B. rapa*, AA; *B. nigra*, BB; *B. oleracea*, CC; *B. juncea*, AABB; *B. napus*, AACC; *B. carinata*, BBCC), the protein sequences of Arabidopsis ICE1 and ICE2 were used as queries to search the Brassicaceae Database (BRAD; http://brassicadb.cn). Dicotyledon tomatoes (*Solanum lycopersicum*), soybeans (*Glycine max*), monocotyledon maize (*Zea mays*), foxtail millet (*Setaria italica*) and rice (*Oryza sativa*) served as controls for comparison with known ICE1 homologs. Phylogenetic analysis revealed distinct clusters for monocot and dicot ICE1 homologs (Figure 1). A total of 42 *ICE1* homologous genes were identified in the six widely cultivated Brassica species, including a variant of *B. rapa* (Z1, yellow sarson, an oilseed crop). These genes were divided into two subgroups (*ICE1* and *ICE2*) based on the phylogenetic tree (Figure 1).

Four ICE1-like homologous genes were each identified in the diploid species *B. rapa* (AA; Chinese cabbage) and *B. oleracea* (CC). Notably, the hybridized allotetraploid species *B. napus* (AACC) contained 10 *ICE1* homologous genes. The diploid *B. nigra* (BB) contained 5 *ICE1* homologous genes, while the hybridized allotetraploid *B. juncea* (AABB) contained 10 *ICE1* homologous genes. The hybridized allotetraploid *B. carinata* (BBCC) contained only 5 *ICE1* homologous genes. Among the three diploid Brassica species, *B. rapa* is a mesohexaploid diploid with a triplicated chromosomally segmented genome [47]. Amino acid sequence alignment of Z1 showed that two *ICE1* homologous genes belonged to the ICE1 subgroup; the other two belonged to the ICE2 subgroup. Notably, four *ICE1* homologous genes were identified in Chiifu-401-42 (*B. rapa*, Chinese cabbage, as a vegetable), among which three belonged to the ICE1 subgroup and one belonged to the ICE2 subgroup. However, BraA06g038100.3C lost a partial domain in the MYC-like bHLH region, suggesting it may not be a true *ICE1* homolog (Supplementary Figure S1). Phylogenetic tree analysis of *ICE1* homologous genes in Brassica species suggested that the evolution of *ICE1* homologous genes was complex during genome hybridization and polyploidization, particularly in allotetraploid Brassica species.

### 2.2. Low Temperatures Induce Diverse Expression Patterns of ICE1 Homologous Genes in Brassica Species

**Figure 2:**
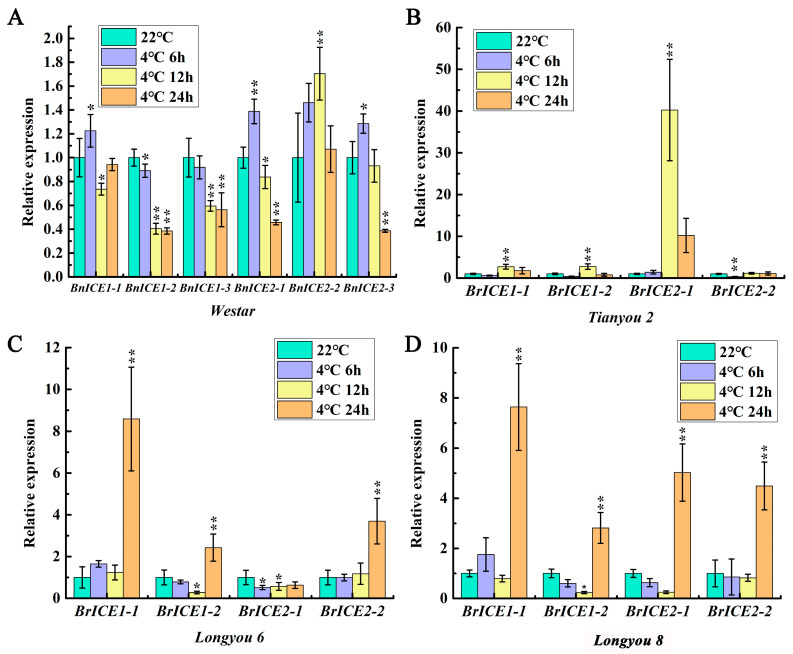
Low temperature induces the expression of *ICE1* homologous genes in Brassica species. The 14-day-old seedlings were low-temperature treated at 4 °C for 6 h, 12 h and 24 h, while the expression levels of *ICE1* homologous genes were determined by qRT–PCR. *BrACTIN2* was used as the control. (**A**) The expression profiles of six *BnICE1* homologous genes in Westar. (**B**–**D**) The expression profiles of four *BrICE1* homologous genes in Tianyou 2, Longyou 6 and Longyou 8. Values are shown as mean ± SD (*n* = 3) of three independent experiments. Statistically significant differences are indicated by asterisks (Student’s *t*-test, *, *p* < 0.05, **, *p* < 0.01).

In different freeze-resistant Arabidopsis thaliana accessions, 4 °C cold treatment significantly induced the expression of *AtICE1*, which peaked at 4 h and then declined. Throughout the 8 h of cold treatment, the expression level was higher in strong freeze-resistant accessions than in weak freeze-resistant accessions [48]. To explore the expression patterns, four freeze-resistant different oilseed rape varieties (Supplementary Figure S2) were used to detect the expression level of *ICE1* homologous genes by quantitative real-time polymerase chain reaction (qRT–PCR). Westar is a *Brassica napus* (2n = 4x = 38, AACC) background, which has weak cold resistance; Longyou 6, Longyou 8 and Tianyou 2 are a *Brassica rapa* (2n = 2x = 20, AA) background. Longyou 6 and Longyou 8 have strong cold resistance, and Tianyou 2 has weak cold resistance. qRT–PCR results showed that low temperatures induced the expression of *ICE1* homologous genes (Figure 2A–D). However, the expression patterns varied across different freeze-resistant varieties. As shown in Figure 2, chilling treatment for 6–12 h induced the expression of *ICE1* homologous genes in Tianyou 2 and Westar (Figure 2A,B). In contrast, Longyou 8 and Longyou 6 displayed significant induction only after 24 h (Figure 2C,D). Moreover, the expression levels of *ICE1* homologous genes peaked at 24 h of chilling stress in Longyou 8 and Longyou 6. However, in Tianyou 2 and Westar, the peak occurred at 6–12 h. These results indicate that the *ICE1* homologous genes in Brassica species are involved in the cold response, and different freeze-resistant varieties exhibited diverse expression patterns.

### 2.3. Cloning and Protein Structural Domain Analysis of BrICE1 Homologous Genes

**Figure 3:**
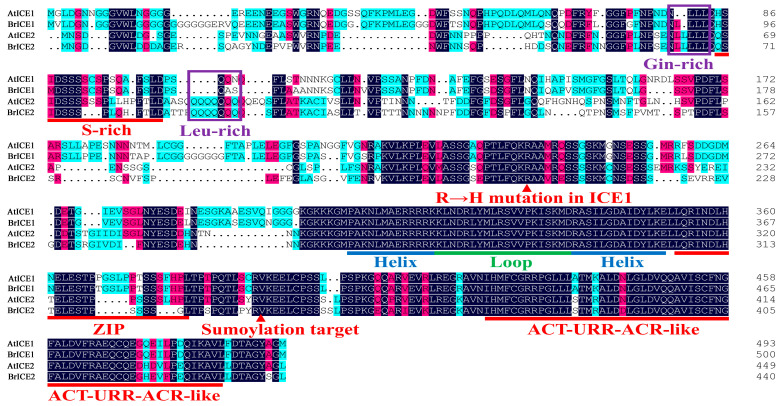
Multiple sequence alignment and domain structure analysis of BrICE1 and BrICE2 of *B. rapa*. The DNAMAN v9.0 software was used to align the amino acid sequences of BrICE1 and BrICE2. Residues in red indicate the conserved serine-rich (S-rich) region sites, ZIP region domain, ICE-specific domain, potential SUMOylation site and ACT-like domains. Residues in blue and green indicate the conserved MYC-like bHLH domain. Residues in purple indicate the specific glutamine-rich (Gin-rich) and leucine-rich (Leu-rich) domains of BrICE2.

Based on the phylogenetic tree and protein structural feature analysis, two of the four *ICE1* homologous genes, *BrICE1-1* and *BrICE2-1*, were isolated from Longyou 6 using reverse transcription PCR (RT–PCR) to investigate their role in cold signaling. The full-length cDNA of *BrICE1-1* contained 1491 bps, encoding a protein of 497 amino acids, whereas the cDNA of *BrICE2-1* comprised 1320 bps, encoding a protein of 440 amino acids (Figure 3). A BLASTp search against BRAD revealed 64.5% identity between BrICE1-1 and BrICE2-1. Protein structural domain analysis of BrICE1-1 and BrICE2-1 demonstrated that they share common structural domains, including a conserved serine-rich (S-rich) region site, zipper (ZIP) region domain, conserved MYC-like bHLH domain, ICE-specific domain, potential SUMOylation site and ACT_UUR_ACR-like (ACT) domain, all of which are typical features of ICE1 [6,9,10]. BrICE2-1 contains a conserved glutamine-rich and leucine-rich region-specific domain in the variable N-terminal (Figure 3). Owing to the *ICE1* homologous genes of *B. rapa* being previously named *BrrICE1.1* (in *Brassica rapa* var. *rapa*) [45] or *BcICE1* (in *Brassica campestris*) [46], to maintain consistent nomenclature for *B. rapa ICE1* homologs, as described previously, *BrICE1-1* and *BrICE2-1* were renamed *BrICE1* and *BrICE2*, respectively.

### 2.4. BrICE1 and BrICE2 Localize to the Nucleus, and Low Temperature Does Not Affect Localization

**Figure 4:**
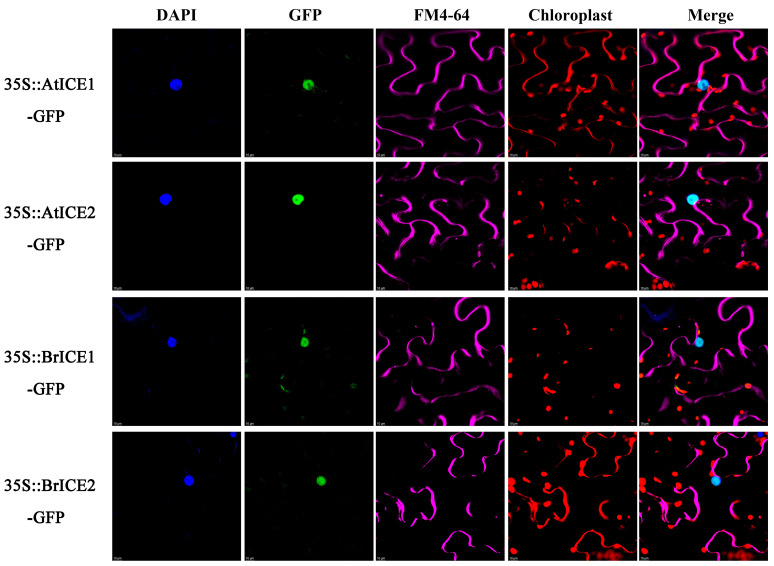
BrICE1 and BrICE2 are nuclear-localized proteins. The 35S:BrICE1-GFP, 35S: BrICE2-GFP, 35S:AtICE1-GFP and 35S:AtICE2-GFP plasmids were constructed and transiently expressed in tobacco leaves. The GFP signal was visualized under a confocal microscope. Nuclei were indicated by 4′,6-diamidino-2-phenylindole (DAPI) staining. Plasma membranes were indicated by FM4-64 (a plasma membrane stain) staining and autofluorescence of chloroplasts was indicated by chlorophyll b staining. From left to right, blue DAPI signal, green GFP signal, purple plasma membranes FM4-64 signal, red chloroplasts autofluorescence signal and merged image signal. Scale bar, 10 μm.

Previous studies have shown that AtICE1 in Arabidopsis functions within the nucleus [6]. To determine the subcellular localization of BrICE1 and BrICE2, 35S:BrICE1-GFP and 35S:BrICE2-GFP were constructed and transiently expressed in tobacco leaves (Figure 4). Meanwhile, Arabidopsis 35S:AtICE1-GFP and 35S:AtICE2-GFP were constructed as the known positive controls. Strong GFP fluorescence of BrICE1-GFP and BrICE2-GFP fusion proteins was observed in the nucleus of transformed cells stained with 4′,6-diamidino-2-phenylindole (DAPI). Similarly, strong GFP fluorescence of AtICE1-GFP and AtICE2-GFP fusion proteins was observed in the nucleus (Figure 4), which is consistent with previous findings [6]. These observations confirm that BrICE1 and BrICE2, similar to AtICE1 in Arabidopsis, localize to the nucleus.

**Figure 5:**
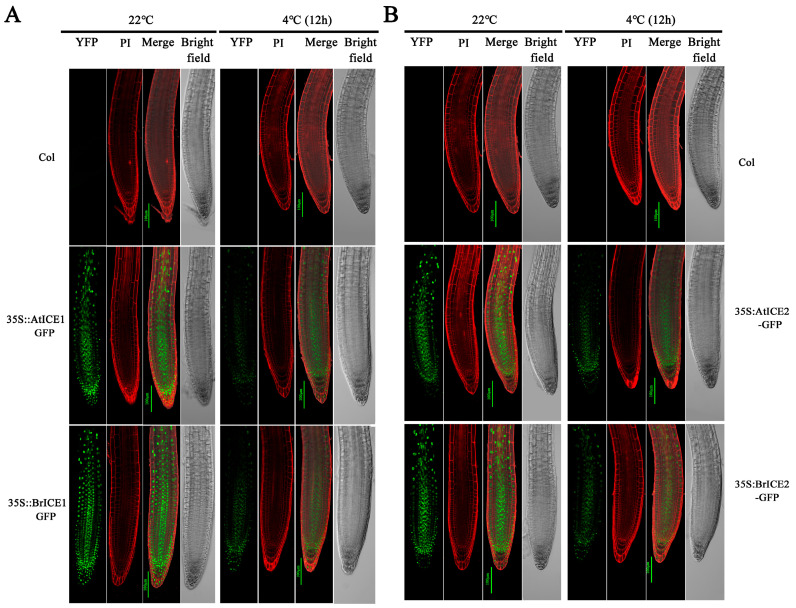
Cold induces the degradation of BrICE1 and BrICE2. The three-day-old seedlings were grown on treated agar plates (4 °C, 12 h), the roots were incubated in 0.02 mg/mL PI for 12 min and the GFP signals in roots were visualized and photographed using confocal microscopy. (**A**) Visualization of AtICE1-GFP and BrICE1-GFP transgenic plants. (**B**) Visualization of AtICE2-GFP and BrICE2-GFP transgenic plants. Scale bar, 100 μm.

Several studies have demonstrated that AtICE1 is mainly localized to the nucleus, and cold stress does not significantly affect its localization [6,13]. To further investigate whether cold stress affects BrICE1 and BrICE2 localization, the roots of *BrICE1-GFP* and *BrICE2-GFP* transgenic plants were observed after cold treatment. Strong GFP fluorescence was observed in the nuclei at 22 °C (Figure 5A,B). While the fluorescence signal remained localized in the nuclei after cold treatment (4 °C for 12 h), it became weaker (Figure 5A,B). Similar changes were observed in the roots of *AtICE1-GFP* and *AtICE2-GFP* transgenic plants. However, we did not observe significant differences between *BrICE1-GFP* and *BrICE2-GFP* transgenic plants. Notably, the fluorescence signal was slightly stronger in *BrICE1-GFP* and *BrICE2-GFP* transgenic plants than in *35S:AtICE1-GFP* and 35S:*AtICE2-GFP*. These results indicate that similar to AtICE1 and AtICE2 in Arabidopsis, BrICE1 and BrICE2 in *B. rapa* are nucleus-localized proteins, and their localization is not affected by cold stress.

### 2.5. BrICE1 and BrICE2 Positively Regulate Cold Tolerance via the CBF-Dependent Pathway in Transgenic Arabidopsis

To further elucidate the role of BrICE1 and BrICE2 in cold resistance, *35S:BrICE1-GFP* and *35S:BrICE2-GFP* were constructed and overexpressed in Arabidopsis. Additionally, *35S:AtICE1-GFP* and *35S:AtICE2-GFP* were overexpressed as positive controls. We obtained 9 and 12 independent transgenic lines from the *BrICE1-OE* and *BrICE2-OE* positive transgenic plants, respectively. The transcriptional and protein levels of six randomly selected T_2_ *BrICE1-OE* and *BrICE2-OE* positive transgenic lines (renamed 1#, 2# and 3#, respectively) were detected by qRT–PCR and Western blot. The results showed that the 1# (*BrICE1-OE1#*, *BrICE2-OE1#*) and 3# (*BrICE1-OE3#*, *BrICE2-OE3#*) transgenic lines maintained stable expression at the transcriptional and protein levels (Supplementary Figure S3). Thus, these transgenic lines were chosen for further analyses. Based on the same selection criteria, stable expressional *AtICE1-OE3#* and *AtICE2 OE3#* transgenic lines were chosen for further analyses.

**Figure 6:**
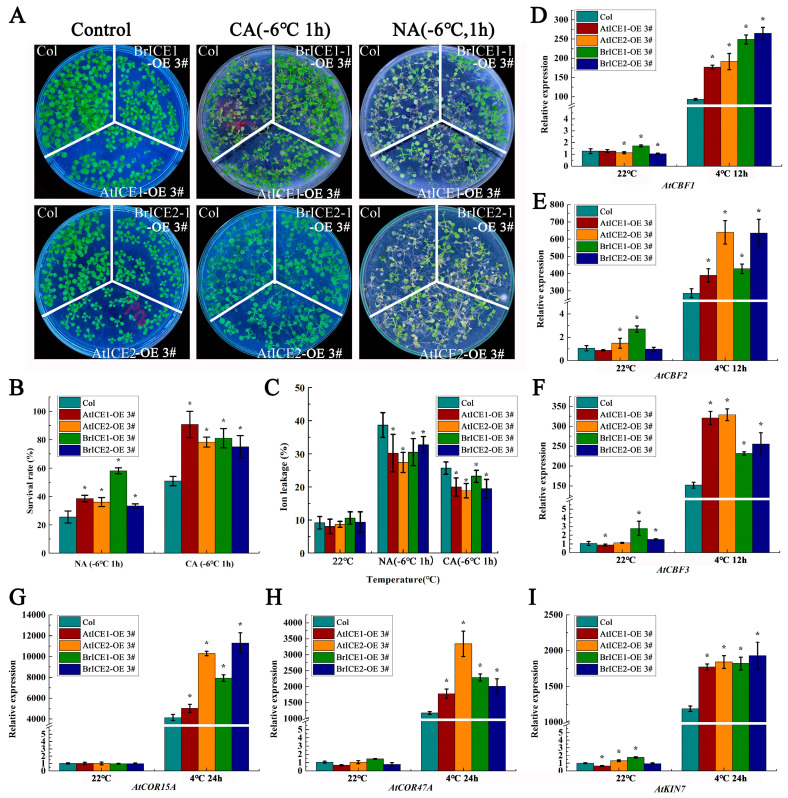
Overexpression of *BrICE1* and *BrICE2* enhances the cold tolerance of transgenic Arabidopsis through the CBF-dependent pathway. The 14-day-old seedlings were subjected to freezing at −6 °C for 1 h with (CA, at 4 °C for 3 days) or without cold accumulation (NA). After 3 days recovery at 22 °C, the survival rates and ion leakage rates were determined. To test the expression levels of CBFs and their target genes, 14-day-old seedlings were low-temperature treated (at 4 °C) for either 12 or 24 h and subjected to qRT–PCR analysis. ACTIN2 was used as the reference gene. (**A**) Freezing phenotypes. (**B**) Survival rates (*n* = 120). (**C**) Ion leakage rates (*n* = 30). (**D**–**F**) Expression levels of *AtCBF1*, *AtCBF2* and *AtCBF3* (*n* = 3). (**G**–**I**) The expression levels of *AtCOR15A*, *AtCOR47A* and *AtKIN7* (*n* = 3). Values are shown as mean ± SD of three independent experiments. Statistically significant differences are indicated by asterisks (Student’s *t*-test, *, *p* < 0.05).

The aerial phenotypes of *BrICE1* and *BrICE2* overexpression lines were not significantly different from the wild-type plants grown at a permissive temperature, except for slightly shorter petioles (Supplementary Figure S3A). The 14-day-old seedlings grown on separate sections of the same agar plates were cold-treated at −6 °C for 1 h with or without cold acclimation (CA, 4 °C for 3 days) before undergoing a freezing tolerance assay. *BrICE1* and *BrICE2* transgenic plants showed significantly enhanced freezing tolerance compared with the wild-type plants under both non-acclimated (NA) and cold-acclimated (CA) conditions (Figure 6A). Without cold acclimation, only 25% of wild-type plants survived after the freezing treatment (−6 °C for 1 h). The survival rate of *BrICE1* transgenic plants was more than 58%, that of *BrICE2* transgenic plants was over 37% and that of *AtICE1* and *AtICE2* transgenic plants was more than 35% (Figure 6B). As expected, cold acclimation not only significantly enhanced the freezing tolerance of transgenic plants but also enhanced the freezing tolerance of wild-type plants. After cold acclimation, approximately 80% of the *BrICE1* and *BrICE2* transgenic plants survived; however, the survival rate of the wild-type plants was only approximately 35% (Figure 6B). Furthermore, under NA conditions, the survival rate of *BrICE1* transgenic plants was significantly higher than that of *BrICE2* plants, suggesting that the overexpression of *BrICE1* conferred stronger resistance to transgenic plants compared with *BrICE2* transgenic plants (Figure 6B).

Subsequent electrolyte leakage assays supported these findings, where ion leakage in *BrICE1* and *BrICE2* transgenic plants was lower than that in wild-type plants (Figure 6C), indicating that cold stress-induced plasma membrane damage was mitigated in *BrICE1* and *BrICE2* transgenic plants. Similar phenotypes were observed in seedlings grown in the soil (Supplementary Figure S4). Consistent with previous reports, the overexpression of Arabidopsis *AtICE1* and *AtICE2* also enhanced cold tolerance [6,10]. As we were unable to obtain loss-of-function T-DNA homozygous lines for *ICE1* and *ICE2* from the Arabidopsis Biological Resource Center (ARBC), we did not conduct complementary experiments in *ice1* and *ice2* loss-of-function mutants. Our observations indicate that BrICE1 and BrICE2 in *B. rapa* function as important positive regulators in response to cold stress, playing overlapping roles with slightly unequal functional redundancy in acquiring freezing tolerance under the present experimental conditions.

AtICE1 in Arabidopsis is involved in the cold stress response by regulating CBF expression [6,49]. To investigate whether BrICE1 and BrICE2 regulate cold signaling through the CBF-dependent pathway, we examined the expression levels of *CBFs* and their target genes, *AtCOR15A*, *AtCOR47* and *AtKIN1*, in transgenic and wild-type plants under cold conditions. As shown in Figure 6, cold stress significantly induced the expression of *CBFs* and their target genes in *BrICE1* and *BrICE2* transgenic and wild-type plants. However, their expression levels were lower in the wild-type plants than in the transgenic plants (Figure 6D–I). Notably, the basal levels of *CBFs* were higher in the *BrICE1* and *BrICE2* transgenic plants than in the wild-type plants, particularly in the *BrICE1* transgenic plants.

### 2.6. Overexpression of BrICE1 and BrICE2 Inhibits Root Growth in Arabidopsis

ICE1 plays an important role not only in cold signaling but also in plant growth and development [8,15,18,19,50,51]. However, the mechanism by which ICE1 balances growth, development and cold-stress response has not been extensively studied. Several studies have showed that constitutive overexpression of *CBFs* adversely affects plant growth under normal growth conditions [52,53], revealing that CBFs are master-regulators of the trade-off between growth and development versus freezing tolerance.

**Figure 7:**
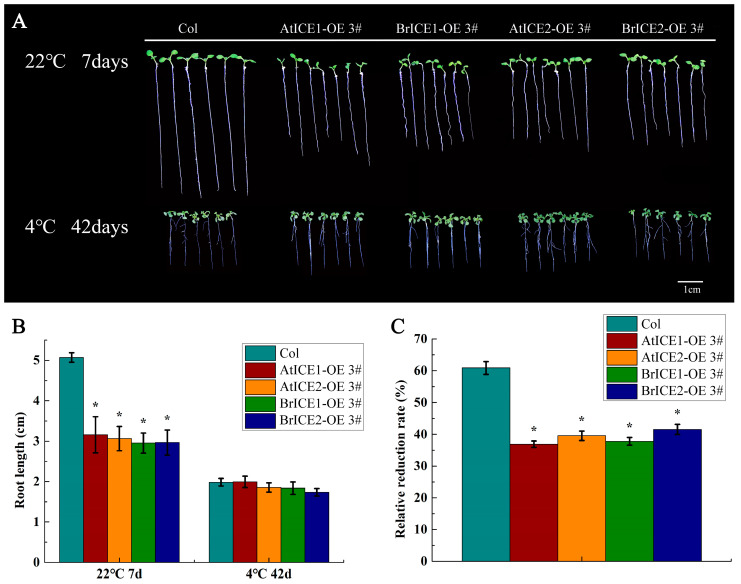
Overexpression *of BrICE1* and *BrICE2* inhibits root growth in Arabidopsis. Seedlings were grown on half-strength MS at 22 °C for 7 days; root length was measured using the Image-Pro Plus 6.0 software and designated as L1. After 3 days at 22 °C, seedlings were cold-treated for 42 days at 22 °C, and root length was measured and designated as L2. The relative reduction rate of root length was calculated as (L1 − L2)/L1 × 100%. (**A**) Root length phenotype. (**B**) Statistical analysis of root length (*n* = 90). (**C**) Relative reduction rate of root length under low-temperature treatment. Values are shown as the mean ± SD of three independent experiments, each with three technical replicates. Statistically significant differences are indicated by asterisks (Student’s *t*-test, *, *p* < 0.05). Scale bar, 1 cm.

To examine whether BrICE1 and BrICE2 can also influence this trade-off, roots growth was analyzed under normal and cold-stress conditions. As shown in Figure 7A, the aerial phenotype of *BrICE1* and *BrICE2* transgenic plants did not exhibit any detectable abnormalities compared with wild-type plants when grown on half-strength Murashige and Skoog (MS) medium at 22 °C. However, root elongation was significantly suppressed in *BrICE1* and *BrICE2* transgenic plants compared with wild-type plants at 22 °C (Figure 7A,B). The root length of wild-type plants was inhibited by approximately 60% when grown at 4 °C for 42 days compared with 22 °C for 7 days. Conversely, the suppression ratio in *BrICE1* and *BrICE2* transgenic plants was only 35% compared with the wild-type plants (Figure 7C). Notably, no significant differences in roots growth were observed between *BrICE1* and *BrICE2* transgenic plants at either 22 °C or 4 °C. Similar results were obtained for the *AtICE1* and *AtICE2* transgenic plants. These results suggest that BrICE1 and BrICE2, similar to CBFs, play critical roles in cold signaling by acting as regulators that balance growth and development with freezing tolerance, potentially through integration with unknown downstream target genes.

### 2.7. Overexpression of BrICE1 and BrICE2 Enhances ROS Scavenging by Elevating Enzymatic Antioxidants in Arabidopsis

**Figure 8:**
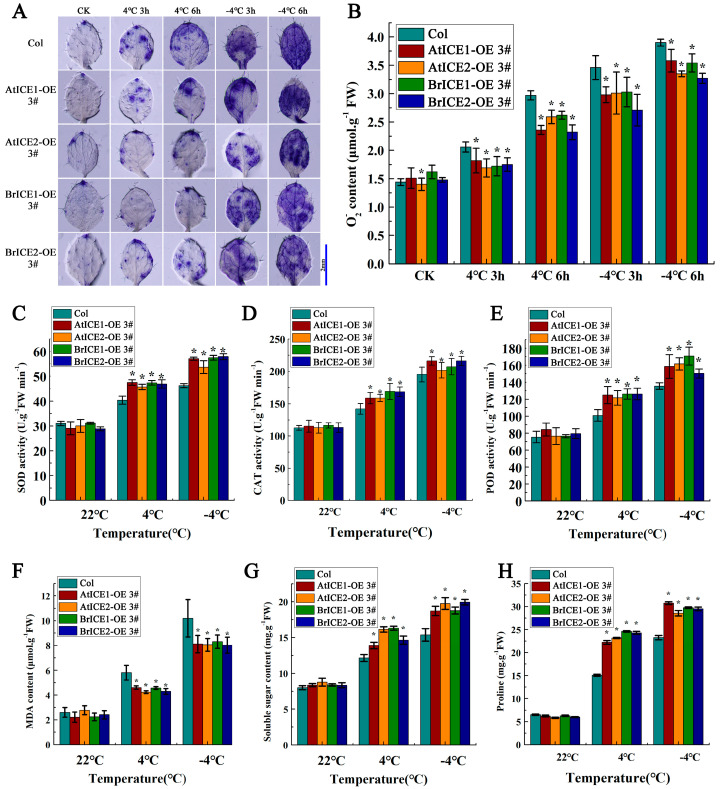
Overexpression of *BrICE1* and *BrICE2* enhances ROS scavenging by elevating enzymatic antioxidants in Arabidopsis. The 10-day-old seedlings were chill- and freeze-treated for either 3 or 6 h, and the leaves were stained using an NBT solution. The phenotype was photographed, and the activities of SOD, CAT, POD and O_2_^−^·, as well as the MDA, soluble sugar and proline contents, were detected. (**A**) The phenotype of ROS accumulation. (**B**) The changes in O_2_^−^· content. (**C**–**E**) The activity of SOD, CAT and POD. (**F**–**H**) The MDA, soluble sugar and proline content. Values are shown as mean ± SD (*n* = 30) of three independent experiments, each with three technical replicates. Statistically significant differences are indicated by asterisks (Student’s *t*-test, *, *p* < 0.05). Scale bar, 2 mm.

Our previous studies have revealed that ROS accumulation and response speed are critical for freezing tolerance in *B. rapa* [42]. To further explore the relationship between BrICE1 and BrICE2 involvement in low-temperature resistance and ROS, we assayed ROS accumulation in *BrICE1* and *BrICE2* transgenic plants using nitroblue tetrazolium (NBT) histochemical staining after chilling (4 °C for 3 or 6 h) or freezing (−4 °C for 3 or 6 h) treatment. Following chilling treatment, the NBT staining intensity was weaker in the leaves of *BrICE1* and *BrICE2* transgenic plants than in the leaves of the wild-type plants, suggesting higher levels of damage after cold stress (Figure 8A). Although freezing stress (−4 °C for 6 h) also caused significant injury to the *BrICE1* and *BrICE2* transgenic plants, the degree of injury was milder compared with that of the wild-type plants. The same phenomenon was observed in the *AtICE1* and *AtICE2* transgenic plants. Quantitative measurements further confirmed these observations, revealing that the superoxide anion (O_2_^−^·) content in *BrICE1* and *BrICE2* transgenic plants was lower than that in wild-type plants under both chilling and freezing stress (Figure 8B). Both histochemical staining and quantitative measurements demonstrated that the overexpression of *BrICE1* and *BrICE2* resulted in lower ROS levels under chilling and freezing stress, suggesting that the overexpression of *BrICE1* and *BrICE2* may confer a more efficient ROS scavenging system in transgenic plants.

To determine whether the rapid ROS scavenging observed in the *BrICE1*- and *BrICE2*-overexpression plants is attributable to enzymatic antioxidative processes, the activities of superoxide dismutase (SOD), catalase (CAT) and peroxidase (POD) were investigated. Under normal conditions, the enzyme activities of SOD, CAT and POD were not significantly different between the *BrICE1* and *BrICE2* transgenic and wild-type plants (Figure 8C–E). However, after chilling (4 °C for 3 h) or freezing (−4 °C for 3 h) treatment, the activities of all three enzymes in the transgenic plants were significantly higher than those in the wild-type plants, with the difference being more significant after the freezing treatment. These results suggest that overexpression of *BrICE1* and *BrICE2* enhances the ROS scavenging ability by increasing the activities of SOD, CAT and POD, potentially contributing to cold tolerance by rapidly balancing ROS accumulation. Furthermore, the malondialdehyde (MDA) content was measured, as shown in Figure 8F. Chilling or freezing stress caused membrane damage, leading to elevated MDA levels. However, the elevation of MDA was lower in *BrICE1* and *BrICE2* transgenic plants than in wild-type plants, suggesting that the overexpression of *BrICE1* and *BrICE2* relieved low-temperature-induced membrane injury.

Plants can increase their tolerance to cold stress by rapidly synthesizing numerous soluble sugar and proline protective substances [54]. Our physiological results showed that *BrICE1* and *BrICE2* overexpression increased the content of soluble sugars and proline compared with those of the wild-type plants after both chilling and freezing treatments (Figure 8G,H). These physiological results suggest that the overexpression of *BrICE1* and *BrICE2* increases cold resistance and is closely correlated with ROS scavenging and osmotic adjustment.

### 2.8. BrICE1 and BrICE2 Are Degraded via the 26S-Proteasome Pathway in Response to the Cold-Stress Pathway

Many studies have confirmed that AtICE1 functions as a transcription activator to activate downstream gene expression in response to cold stress [6,7,9,10,22,25]. To determine whether BrICE1 has transcriptional activity, we used BrOST1 (a well-known Ser/Thr protein kinase [12]) as a target protein to test BrICE1’s transactivation potential. The BrICE1 and BrOST1 of *B. rapa* were cloned into pGBKT7 and pGADT7 vectors, respectively. The yeast two-hybrid results demonstrated that BrICE1 has transcriptional activity and can interact with BrOST1 (Supplementary Figure S5).

**Figure 9:**
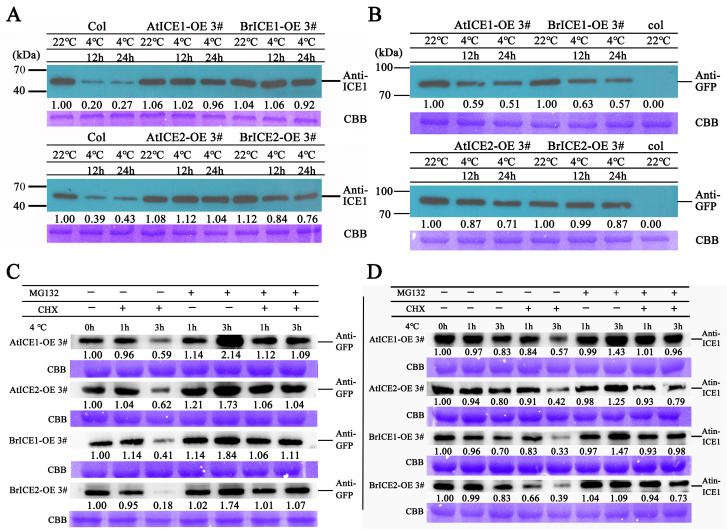
Cold-induced degradation of BrICE1 and BrICE2 depends on the 26S-proteasome pathway. The 14-day-old wild-type and transgenic seedlings were treated at 4 °C for 1 to 24 h with or without 100 mM CHX and 50 mM MG132. Total protein was extracted and immunoblotting was performed using specific anti-ICE1 and anti-GFP antibodies. Coomassie brilliant blue (CBB) was used as the control for protein loading. The integrated optical density (IOD) values of ICE1 bands were quantified. (**A**,**B**) Immunoblotting assays to assess the protein level in wild-type and transgenic seedlings without CHX and MG132 treatment using specific anti-ICE1 (**A**) and anti-GFP (**B**) antibodies. (**C**,**D**) Immunoblotting assays to assess the protein levels in wild-type and transgenic seedlings with CHX and MG132 treatment using specific anti-ICE1 (**A**) and anti-GFP (**B**) antibodies.

Accumulating evidence suggests that ICE1 functions in cold signaling through PTMs [4]. To investigate whether BrICE1 and BrICE2 also function in cold tolerance through PTMs, BrICE1 and BrICE2 protein levels were determined in transgenic and wild-type plants before and after cold treatment using specific anti-ICE1 (specific for AtICE1 and BrICE1) and anti-GFP antibodies. As shown in Figure 9A, in wild-type plants, a 12 h cold treatment at 4 °C induced a substantial reduction in endogenous ICE1 protein abundance. However, the total protein levels of BrICE1 (using the specific anti-ICE1 antibody to test) and transgenic protein levels of BrICE1 and BrICE2 (using an anti-GFP tag antibody) in the *BrICE1* and *BrICE2* transgenic plants did not decrease significantly until after 24 h of low-temperature treatment (Figure 9A,B). This phenomenon was also observed in *AtICE1* and *AtICE2* transgenic plants. It is worth noting that the overexpression of BrICE1 and BrICE2 slightly elevated the normal thermal endogenous protein abundance of AtICE1. To determine whether the overexpression of *BrICE1* and *BrICE2* leads to an increase in endogenous AtICE1, the transcription level was tested using specific AtICE1 and AtICE2 primers. The results showed that only the overexpression of *AtICE2* increased the endogenous transcriptional level of AtICE1, and not the overexpression of *BrICE1* and *BrICE2* (Supplementary Figure S6), suggesting that the overexpression of *BrICE1* and *BrICE2* did not affect the expression of *AtICE1*, at least on the transcriptional level.

Next, the anti-GFP antibody was used to assess the fusion protein levels of *BrICE1-GFP* and *BrICE2-GFP* in transgenic plants after cold treatment. The results show that cold treatment induced a substantial reduction in both BrICE1-GFP and BrICE2-GFP fusion proteins, with the reduction in the BrICE2-GFP fusion protein being lower than that of BrICE1-GFP. Similarly, the AtICE2-GFP fusion protein reduction was lower than that of AtICE1-GFP in transgenic plants after cold treatment (Figure 9B). These Western blot results are consistent with the observed root phenotypes in the nuclear localization experiment, suggesting that, similar to AtICE1 in Arabidopsis, low temperatures can also induce the degradation of BrICE1 and BrICE2 in vivo.

Furthermore, the stability of BrICE1-GFP and BrICE2-GFP fusion proteins was investigated in the presence of MG132 (a 26S-proteasome inhibitor) and cycloheximide (CHX, a protein synthesis inhibitor) using anti-GFP and specific anti-ICE1 antibodies. As shown in Figure 9, low temperature obviously induced the degradation of BrICE1 and BrICE2 proteins, but this degradation could be dramatically blocked by MG132 (Figure 9C,D). It is noteworthy that the degradation of ICE1 was less pronounced in *BrICE1* and *BrICE2* transgenic plants than it was in *AtICE1* and *AtICE2* transgenic plants. Interestingly, the cold-induced degradation of BrICE1 and BrICE2 proteins was weaker when using the specific anti-ICE1 antibody (Figure 9D) than when using the anti-GFP antibody (Figure 9C). Regardless, these data suggest that in *B. rapa*, the low-temperature-induced degradation of BrICE1 and BrICE2 occurs via the 26S-proteasome pathway.

## 3. Discussion

Several studies have reported that some *B. rapa* varieties can survive extremely low temperatures (down to −32 °C) during overwintering [30,55]. However, the underlying molecular mechanisms remain unclear. The ICE1–CBF–COR regulatory cascade is regarded as the most essential cold-signaling pathway in Arabidopsis, with ICE1 acting as a crucial regulator. Consequently, several *ICE1* paralogs from Brassica species have been cloned and characterized [45,46,56]. However, the phylogeny and roles of ICE1 and ICE2 in *B. rapa* cold signaling have not been extensively investigated.

### 3.1. ICE1 Homologs Exhibit High Conservation across Brassica Species

In this study, four *ICE1* paralogs were identified in Z1 (*B. rapa*, yellow sarson) and three in Chiifu-401-42 (*B. rapa*, Chinese cabbage). Although both Chiifu-401-42 and Z1 are diploid *B. rapa* varieties, the number of *ICE1* and *ICE2* paralogs differed between them. This discrepancy suggests a complex evolutionary process within *B. rapa* species. One potential explanation is that ICE2 in Arabidopsis arose from a recent duplication event within the Brassicaceae family, estimated at around 17.9 million years ago [9]. Additionally, Z1 may represent a variant within *B. rapa* crops. This hypothesis is supported by the phylogenetic analysis of ICE proteins in *B rapa*. In Chiifu-401-42, two *BraICE1* paralogs reside on chromosomes 2 and 9, while a single *BraICE2* paralog is located on chromosome 8. Similarly, Z1 possesses two *BrICE1* paralogs on chromosomes 2 and 6, with two additional *BrICE2* paralogs on chromosomes 6 and 8. These findings suggest a convoluted process of paralogous gene selection on different chromosomes between Chiifu-401-42 and Z1, potentially reflecting a complex evolutionary mechanism in other cultivated Brassica species.

BrICE1 and BrICE2 amino acid sequences exhibited high conservation with their homologous genes in Brassica species. However, the identity between BrICE1 and BrICE2 was only 64.5%, implying an unequal evolutionary event. Gene duplication events can lead to the retention of some transcripts [57]. Paralogous genes, such as *BrICE1* and *BrICE2*, may persist after undergoing subfunctionalization or neofunctionalization, or experiencing gene dosage effects [56,57,58,59]. In Arabidopsis, ICE2 presumably originated from a duplication event in early Brassicaceae species approximately 17.9 million years ago. This was followed by the sequence and functional diversification of ICE1 [9]. The duplication and subsequent subfunctionalization of BrICE2 might explain the low sequence identity observed between BrICE1 and BrICE2.

Brassica species belong to the Brassicaceae family, with 3700 known species across 340 genera [43], which include three diploid species (*B. rapa*, *B. nigra* and *B. oleracea*) and three amphidiploid species (*B. juncea*, *B. napus* and *B. carinata*). The complex history of genome hybridization and polyploidization within this family has resulted in intricate genomic information among Brassica species, often referred to as “U’s triangle” [44]. For instance, the diploid species *B. rapa* (Chiifu-401-42) and *B. oleracea* contain three and four *ICE1* paralogs, respectively, while their allotetraploid offspring, *B. napus,* contains ten *ICE1* homologous genes (Figure 1). Owing to the limited scope of the present research, definitively elucidating the evolutionary relationship between *ICE1* and *ICE2* in Brassica species remains challenging.

ClustalW protein sequence alignment revealed the presence of glutamine- and leucine-rich region domains in BrICE2 (Figure 1), similar to those found in Arabidopsis ICE2 [9]. This suggests a conserved evolutionary trajectory for ICE2 in Brassica species. Future studies will investigate whether these specific domains govern unknown physiological functions in BrICE2. Therefore, our study identified 41 ICE1-like homologous genes in six widely cultivated Brassica species, distinguishing between ICE1 and ICE2. The gene structure of *ICE*s is highly conserved in Brassica species; however, their gene duplication events are complicated.

### 3.2. Overexpression of BrICE1 and BrICE2 in Arabidopsis Enhances Cold Tolerance through CBF and ROS Scavenging Pathways

Over the past two decades, research has established that ICE directly binds to CBF promoters, regulating the cold-signaling cascade, a key regulating mechanism of ICE1 in many species [20,21,22,23,24,28]. Our expression pattern analysis revealed that low temperatures induced the expression of *ICE1* homologous genes in all tested varieties (Figure 2). However, the expression patterns differed between freeze-resistant varieties. Strongly cold-tolerant varieties required longer low-temperature stress periods to activate *ICE1* expression compared to weakly cold-resistant varieties. We speculated that strongly cold-resistant varieties might have stronger basal cold resistance than weakly cold-resistant varieties. Under low-temperature stress, strongly cold-resistant varieties may not require promoting *ICE1* expression until basal cold resistance is exhausted. Conversely, varieties with weaker cold resistance need to promote *ICE1* expression earlier, owing to their weaker basal cold resistance. This regulated molecular mechanism requires further investigation.

*BrICE1* and *BrICE2*, isolated from the strongly cold-resistant variety Longyou 6 (Supplementary Figure S2), were found to localize to the nucleus (Figure 4), similar to *AtICE1* and *AtICE2* of Arabidopsis. This localization was not affected by cold stress (Figure 5), consistent with a previous study in Arabidopsis [6]. The cold-activated upregulation of the expression of *CBFs* and their target genes (*AtCOR15A*, *AtCOR47* and *AtKIN1*) was higher in transgenic plants than in wild-type plants (Figure 6), suggesting that the overexpression of *BrICE1* and *BrICE2* enhanced cold resistance and was dependent on the CBF-signaling pathway. Additionally, the expression level of *CBFs* was also obviously higher in the *BrICE1* transgenic plants than in the wild-type plants, even without cold accumulation. A detail analysis found that the elevated expression of *CBFs* in *BrICE1* transgenic plants might come from the gene expressive abundance of BrICE1. Moreover, the qRT–PCR results also suggested that the overexpression of *BrICE1* and *BrICE2* led to enhanced cold tolerance not because it induced endogenous *AtICE1* expression, but because it overexpressed *BrICE1* and *BrICE2* themselves. Notably, the survival rate of BrICE2-OE#3 with and without cold acclimation is slightly higher than of BrICE1-OE#3 when grown in soil. The discrepancy in Figure 6B and Figure S4 might be due to different conditions for plant growth.

These results collectively indicate that *BrICE1* and *BrICE2* are novel putative *ICE1* homologs in *B. rapa* and that cold tolerance is also dependent on the CBF pathway. While BrrICE1.1 in *B. rapa* var. *rapa* [45] can directly bind to the promoter of BrrADC2.2, positively regulating its expression and response to cold stress, this suggests that some *ICE1* homolog genes might also be involved in non-CBF-dependent pathways under cold stress. Our results further demonstrate that *BrICE1* or *BrICE2* overexpression could elevate the ROS scavenging ability via enzymatic antioxidative processes and increase the accumulation of proline and soluble sugars in response to cold stress (Figure 8). These findings suggest that BrICE1 and BrICE2 may have evolved multiple regulatory mechanisms to adapt to environmental stress.

Previous studies demonstrated that ICE1-mediated cold tolerance requires a period of cold acclimation. For example, the overexpression of *Hevea brasiliensis HbICE1* and wheat *TaICE87/41* in Arabidopsis enhanced freezing tolerance only after cold acclimation [20,60]. However, our data indicate that BrICE1 might play a role in both cold acclimation-dependent and basal freezing tolerance. *BrICE1* transgenic plants exhibited significantly higher survival rates compared with wide-type plants, even without cold acclimation. This discrepancy may be due to the functional differentiation of ICE1 in different species. In Arabidopsis, AtICE1 and AtICE2 play overlapping roles in cold signaling, but ICE1 plays a predominant role [11].

### 3.3. BrICE1 and BrICE2 Balance Development and Cold Defense

ICE1 is not only a central component of the ICE1–CBF–COR cold-signaling pathway but also serves as a convergence point, integrating multiple signals to regulate cold tolerance and plant growth development. Our root growth assay revealed that the overexpression of *BrICE1* and *BrICE2* suppressed root growth under normal conditions but not under cold stress (Figure 7). This suggests that BrICEs function as a positive regulatory factor that balances plant defense and development. Under a constant energy supply, the overexpression of *ICE1* enhances cold tolerance, which requires more energy. Consequently, less energy is distributed for development. This observation is supported by a study where the overexpression of *CBF1* or *CBF2* in transgenic plants resulted in smaller stature, slower growth rates and a more prostrate growth habit compared with wild-type plants [50]. These findings reveal that BrICE1 and BrICE2 function as network nodes, integrating different signals to regulate cold tolerance and root growth in *B. rapa.* However, the detailed molecular mechanisms underlying this regulation require further investigation.

### 3.4. Post-Translational Modifications Are Crucial for BrICE1 and BrICE2 Response to Cold Stress

ICE1-regulated cold tolerance involves the activation of downstream CBFs and their target *COR* genes [6,61]. However, *AtICE1* is constitutively expressed and can slightly upregulate expression by cold stress [6], suggesting that PTM mechanisms play a crucial role in ICE1 function during cold signaling. Furthermore, studies have shown that ubiquitination and SUMOylation regulate ICE1 stability, allowing plants to balance growth and development under cold stress [14]. Our immunohistochemical analysis and bimolecular fluorescence results revealed that cold induced the degradation of BrICE1 and BrICE2 (Figure 5 and Figure 9). This suggests that similar to AtICE1, BrICE1 and BrICE2 rely on PTM mechanisms for their involvement in cold tolerance. This conclusion is further supported by our yeast two-hybrid assay of BrICE1 and BrOST1 (Supplementary Figure S5). OSTI interacts with ICE1, stabilizing it by preventing its degradation [12]. This reduced degradation observed in *BrICE2* transgenic plants was lower than that in *BrICE1* transgenic plants because of the unequal functional redundancy between BrICE1 and BrICE2 in cold signaling, albeit with varying degrees of effectiveness. Similar results were observed in *AtICE1* and *AtICE2* transgenic plants. In summary, our findings suggest that *BrICE1* and *BrICE2* function as *ICE1* paralogs in *B. rapa*, similar to *AtICE1* and *AtICE2*, and that their roles in cold signaling involve PTM.

## 4. Materials and Methods

### 4.1. Plant Materials and Growth Conditions

The Arabidopsis thaliana ecotype Col-0 and transgenic seedlings used in this study were grown on half-strength MS medium supplemented with 1% sucrose and 0.8% agar at 22 °C under a 16 h light/8 h dark photoperiod. For soil growth, Arabidopsis and *B. rapa* seeds (Weatar, Tianyou 2, Longyou 6 and Longyou 8) were vernalized at 4 °C for 3 days and then grown at 22 °C in a greenhouse under a 16 h light/8 h dark cycle [31,42].

### 4.2. Identification and Phylogenetic Analysis of ICE1 Homologous Genes

To identify ICE1 homologs in six widely cultivated Brassica species (*B. rapa*, AA; *B. nigra*, BB; *B. oleracea*, CC; *B. juncea*, AABB; *B. napus*, AACC; *B. carinata*, BBCC), the amino acid sequences of Arabidopsis ICE1 (At3g26744) and ICE2 (At1g12860) were used as queries to search against BRAD (http://brassicadb.cn) with an e-value threshold of 1e−05 and maximum identity of 50%. Pfam (http://pfam-legacy.xfam.org/, accessed on 18 June 2022) and the National Center for Biotechnology Information (NCBI) Conserved Domain Database (https://www.ncbi.nlm.nih.gov/Structure/cdd/cdd.shtml, accessed on 28 May 2022) were used to evaluate the conserved domains of ICE1 homologs, and redundant sequences were removed. Dicotyledon tomato, soybean (*Glycine max*), monocotyledon maize (*Zea mays*), foxtail millet (*Setaria italica*) and rice (*Oryza sativa*), known to possess ICE1-like homologous genes, were searched in the Phytozome (https://phytozome-next.jgi.doe.gov/, accessed on 12 May 2022) public database as controls. The DNAMAN v9.0 software (Lynnon Corporation, San Ramon, CA, USA) was used to align the amino acid sequences. The MEGA 6.0 software (Molecular Evolutionary Genetics Analysis, The Pennsylvania State University, University Park, PA, USA) [62] was employed to construct a phylogenetic tree based on the full-length protein sequences of ICE1 homologous genes.

### 4.3. Plant Freezing Tolerance and Physiological Assays

Arabidopsis freezing tolerance and physiological assays were performed as previously described [42]. For the NA treatment, 14-day-old seedlings grown on a half-strength MS medium were directly subjected to a freezing chamber for the freezing assay, as described in the figure legends. For the CA treatment, 14-day-old seedlings were pre-treated at 4 °C for 3 days before the freezing assay, as described in the figure legends. After the freezing treatment (specific time and temperature details provided in the figure legends), the seedlings were kept at 4 °C for 12 h and then recovered for 72 h at 4 °C. Subsequently, the survival rates [63]; ion leakage [64]; POD [65], SOD [66] and CAT activities; and MDA content [67] were determined.

The freezing tolerance assays of soil-grown seedlings were similar to those described above. Briefly, 35-day-old Arabidopsis and 12-day-old *B. rape* seedlings with or without cold-acclimation were subjected to a freezing assay, as described in the figure legends. After freezing treatment, the seedlings were kept at 4 °C for 12 h under darkness, and then recovered at 22 °C for 72 h under a 16 h light/8 h dark cyclic photoperiod. The phenotypic, survival and ion leakage rates were counted.

### 4.4. RNA Preparation and qRT–PCR Assays

Total RNA was extracted from Arabidopsis and *B. rapa* seedlings using an RNAprep Pure Plant Kit (No. PD423, TIANGEN, Beijing, China) with or without freezing treatment as described in the figure legends. cDNA was synthesized using the Hifair^®^ Ⅱ 1st Strand cDNA Synthesis Kit (No. 11120ES60, YEASEN, Shanghai, China). qRT–PCR was performed using SYBR Green Master Mix (No. 11202ES08, YEASEN, Shanghai, China) on a QuantStudio™ 5 System. Arabidopsis and *B. rapa ACTIN2* were used as reference genes. Primers used for qRT–PCR are listed in Supplementary Table S1.

### 4.5. Gene Cloning and Plasmid Construction

Full-length cDNA fragments of *BrICE1*, *BrICE2*, *AtICE1* and *AtICE2* were cloned by RT–PCR and transferred into the plant expression vector pBIB-BASTA-35S-GWR-GFP [68] using gateway technology. The expression vector containing the target gene fragments was transformed into the *Agrobacterium* GV3101 recombination strain. Arabidopsis plants were transformed using the floral dip method [69]. Furthermore, T_1_ seedlings were screened on 0.1% (*v*/*v*) basta in the soil, and T_2_ transgenic plants were verified by qRT–PCR and Western blotting with anti-GFP antibodies (No. 1181446001, Roche, Basel, Switzerland). All primers used for cloning and qRT–PCR analyses are listed in Supplementary Table S1.

### 4.6. GFP Fluorescence Assay

Subcellular localization of BrICE1 and BrICE2 was determined as previously described [42]. Briefly, the full-length cDNA of BrICE1, BrICE2, AtICE1 and AtICE2 were amplified by PCR and inserted into the pBIB-BASTA-35S-GWR-GFP vector, and transformed into an *Agrobacterium* GV3101 recombinant strain. Following incubation at 28 °C for 18–20 h, the *Agrobacterium* cultures were injected into tobacco leaves. These tobacco plants were then kept at 22 °C in darkness for 12 h, followed by 22 °C under light conditions for 48 h. Then, a portion of the leaves was excised and incubated in a 4′,6-diamidino-2-phenylindole (DAPI; 500 mM) and FM4–64 (500 mM) solution for 10–15 min. GFP fluorescence was visualized under a confocal microscope (TCS SP8, Leica, Wetzlar, Germany). GFP and chlorophyll b were excited using a 488 nm laser, and detected at 500–550 and 664–696 nm, respectively. FM4-64 were laser-excited at 532 nm, and were detected at 640–660 nm. DAPI were excited at 405 nm and the emission signal was collected between 420 and 440 nm.

Protein degradation assays for BrICE1 and BrICE2 under cold stress were performed as previously described [42]. The roots of 3-day-old seedlings were incubated in 0.02 mg/mL propidium iodide (PI) solution for 12 min. The GFP signal in the roots was then visualized and photographed using a confocal microscope. To verify the cold-induced degradation of BrICE1 and BrICE2, 3-day-old wild-type and transgenic seedlings were incubated at 4 °C for 12 h. Subsequently, the GFP signal in the roots was visualized and photographed. The PI signal was laser-excited at 488 nm, and detected at 630 nm.

### 4.7. Root Growth Inhibition Assays

Arabidopsis thaliana ecotype Col-0 and transgenic seedlings were grown on half-strength MS at 22 °C for 7 days under a 16 h light/8 h dark photoperiod. Root length was measured using ImageJ software (National Institutes of Health, Bethesda, MD, USA) and designated as L1. To test the growth development of roots under cold stress, Arabidopsis Col-0 and transgenic seedlings were grown on half-strength MS at 22 °C for 3 days with a 16 h light/8 h dark photoperiod. These seedlings were then grown at 4 °C for an additional 42 days under the same photoperiod. Root length was subsequently measured as L2. The relative reduction rate in root length was calculated as (L1 − L2)/L1 × 100%.

### 4.8. Histochemical Staining and O_2_^−^· Detection of ROS

Histochemical staining and O_2_^−^· detection were performed as previously described [42]. Notably, 4-day-old seedlings were subjected to chilling (4 °C, 3 or 6 h) and freezing (−4 °C, 3 or 6 h) treatments. Their leaves were then incubated in an NBT solution (Med Chem Express, 0.1% NBT in 10 mM sodium azide and 10 mM phosphate buffer, pH 7.8) overnight. The next day, these leaves were decolorized with 95% ethanol 3–4 times and photographed. The O_2_^−^· content was detected as previously described [42].

### 4.9. Yeast Two-Hybrid Assays

BrOST1, a protein kinase known to interact with ICE1, was cloned into the pGBKT7 vector as a positive control. BrICE1 was cloned into the pGADT7 vector, and both constructs were co-transformed into the yeast strain AH109. Yeast cells were grown on synthetic complete (SC) medium lacking leucine and tryptophan (SC-Leu-Trp) or SC-Leu-Trp-His-Ade medium supplemented with 2 mM 3-amino-1,2,4-triazole (3-AT) for 5 days at 30 °C. Growth on the medium containing 3-AT indicates an interaction between BrICE1 and BrOST1.

### 4.10. Protein Extraction and Immunoblotting Assays

Total protein extraction and immunoblotting were performed as previously described [31]. For transgenic plant authenticity testing, immunoblot analysis was performed using an anti-GFP antibody to detect GFP-fusion proteins; Coomassie brilliant blue (CBB) was used as the control for protein loading.

For the protein degradation assay, 14-day-old wild-type and transgenic seedlings were treated according to the specific time and temperature conditions described in the figure legends. Total protein was then extracted and subjected to immunoblot analysis. ICE1 protein was detected using a specific anti-ICE1 antibody (No. AS16 3971, Agrisera, Vannas, Sweden). The ICE1-GFP fusion protein was detected with an anti-GFP antibody; Coomassie brilliant blue (CBB) was used as the control for protein loading.

To verify whether the low-temperature-induced degradation of BrICE1 and BrICE2 was dependent on the 26S-proteasome pathway, 14-day-old wild-type and transgenic seedlings were treated with or without 100 mM CHX and 50 mM MG132, as described in the figure legends. Then, total protein was subsequently extracted and subjected to immunoblot analysis, as described above. The Image-Pro Plus6.0 software (Media Cybernetics, Rockville, MD, USA) was used to quantify the integrated optical density (IOD) values of ICE1 and actin bands.

### 4.11. Statistical Analysis

All statistical analyses and qRT–PCR experiments were repeated in at least three independent experiments, each with three technical replicates. Data were analyzed using IBM SPSS Statistics 26.0 (IBM Corporation, Armonk, NY, USA) and are presented as the mean ± SD. Significance tests were performed using Student’s *t*-tests (*, *p* < 0.05).

## 5. Conclusions

In this study, 41 ICE1-like homologous genes were identified in six widely cultivated Brassica species. These ICE1-like homologs exhibit high conservation in Brassica species, but their gene duplication events are complicated. Low temperatures induced expression patterns of *ICE1* homologs that differed between freezing-resistant varieties. Two cloned novel ICE1 paralogs of *B. rapa*, *BrICE1* and *BrICE2*, were identified and found to be nuclear-localized proteins; their localization was not affected by cold stress. BrICE1 and BrICE2 positively regulated cold tolerance via the CBF-dependent pathway and ROS scavenging mechanism; these regulating mechanisms are also responsible for balancing the development and cold defense of *B. rapa*.

## Data Availability

The data presented in this study are available on request from the corresponding author.

## Supplementary Materials

The following supporting information can be downloaded at https://www.mdpi.com/article/10.3390/plants13182625/s1, Figure S1. Amino acid alignment of 42 ICE1 homologous genes; Figure S2. The phenotypic and physiological analysis of *B. rapa* under different freezing treatments; Figure S3. Overexpression of BrICE1 and BrICE2 Arabidopsis phenotype and expression level analysis; Figure S4. Overexpression of BrICE1 and BrICE2 enhances the cold tolerance in Arabidopsis; Figure S5. BrICE1 transcription activity analysis; Figure S6. Detection of endogenous AtICE1 and AtICE2 expression under cold-induced conditions. Table S1. Overview of the primers used for cloning, and qRT-PCR.
