# Supplementary material for: Brassica rapa BrICE1 and BrICE2 Positively Regulate the Cold Tolerance via CBF and ROS Pathways, Balancing Growth and Defense in Transgenic Arabidopsis
Source: Plants (Basel). 2024 Sep 20;13(18):2625. doi: 10.3390/plants13182625 (PMC11435425; doi:10.3390/plants13182625)
Supplement: Supplementary file 1 [file plants-13-02625-s001.zip › Supplementary Figure Legends.pdf]

Article

Brassica rapa BrICE1 and BrICE2 Positively Regulate the Cold Tolerance via CBFs and ROS Pathways, Balancing Growth and Defense in Transgenic Arabidopsis

Wangze Wu [1,3,†,*], Haobo Yang [2,†], Peng Xing [1], Guoting Zhu [1], Xueyan Han [1], Mei Xue [1], Guotai Min [1], Haijun Ding [1], Guofan Wu [1] and Zigang Liu [3]

[1] College of Life Sciences, Northwest Normal University, Lanzhou 730070, China; xxfxpxp@163.com (P.X.); zhuguo2022212@163.com (G.Z.); 2021212777@nwnu.edu.cn (X.H.); 2023212787@nwnu.edu.cn (M.X.); hh7777rose@163.com (H.D.); 202131901405@nwnu.edu.cn (G.M.); wugf1971@163.com (G.W.)

[2] Guangdong Provincial Key Laboratory of Plant Adaptation and Molecular Design, School of Life Sciences, Guangzhou University, Guangzhou, 510006, China; yhb998125@163.com (H.Y.)

[3] State Key Laboratory of Aridland Crop Science, College of Agronomy, Gansu Agricultural University, Lan-zhou 730070, China; lzgworking@163.com (Z. L.)

* Correspondence: wangzew78@sina.cn (W.W.);

† These authors contributed equally to this work.

Figure Legends

Supplementary Figure S1. Amino acid alignment of 42 ICE1 homologous genes.
DNAMAN v9.0 software was used to align the amino acid sequences, and protein structural domain analysis. Conserved glutamine-rich and leucine-rich region of *ICE2* homologous genes were signed.

Supplementary Figure S2. The phenotypic and physiological analysis of *B. rapa* under different freezing treatments.
Twelve-day-old *B. rape* seedlings with (CA) or without (NA) cold-acclimated were freezing treated at -7 ℃ for 2 h. After freezing treatment, the seedlings were kept at 4°C for 12 h in darkness, and then were recovered at 22 ℃ for 72 h under normal condition. The phenotypic, survival rates, and ion leakage rates were counted. **(A)** The freezing phenotype. **(B)** The survival rates. **(C)** The ion leakage.

Supplementary Figure S3. Overexpression of BrICE1 and BrICE2 Arabidopsis phenotype and expression levels analysis.
BrICE1-GFP, BrICE2-GFP, AtICE1-GFP and AtICE2-GFP gene were drove by 35S promoter, and were overexpressed in Arabidopsis. The transcription and protein expression levels of twelve randomly selected T_2 transgenic lines were detected by qRT-PCR and western bolt. **(A)** The phenotype of transgenic plants. **(B)** qRT-PCR detects the transcriptional levels of twelve transgenic lines. **(C, D)** western bolt detects the protein levels of twelve transgenic lines by anti-GFP antibody.

Supplementary Figure S4. Overexpression of BrICE1 and BrICE2 enhances the cold tolerance in Arabidopsis.
Twenty-eight-day-old seedlings that grown in soil were freezing treated at - 6 ℃ for 6 h with (CA, at 4 ℃ for 3 d) or without cold accumulation (NA), after 5 d recovering at 22 ℃, the freezing-

phenotype and survival rates were determined.

Supplementary Figure S5. BrICE1 transcription activity analysis.
The full-length of BrICE1and BrOST1 were fused into pGBKT7 and pGADT7, respectively, and were expressed in the yeast strain AH109.

Supplementary Figure S6. Detection of endogenous AtICE1 and AtICE2 expression under cold-induced conditions.
Fourteen-day-old wild-type and transgenic seedlings were treated at 4 °C for 12 to 24 h. Arabidopsis AtICE1- and AtICE2-specific sequence primers were used to detect the transcriptional of endogenous AtICE1 and AtICE2 in wild type and transgenic plants under cold treatment.
Supplementary Table S1. Overview of the primers used for cloning, and qRT-PCR.
