# Supplementary material for: Brassica rapa BrICE1 and BrICE2 Positively Regulate the Cold Tolerance via CBF and ROS Pathways, Balancing Growth and Defense in Transgenic Arabidopsis
Source: Plants (Basel). 2024 Sep 20;13(18):2625. doi: 10.3390/plants13182625 (PMC11435425; doi:10.3390/plants13182625)
Supplement: Supplementary file 1 [file plants-13-02625-s001.zip › Supplementary Figure S1.pdf]

```
AtICE1      ..........................................MGL   3
BrICE1-1    .......MLIHMTYHITINDGNEEKAKKVTRNLNCEKALTFTMVL  38
BrICE1-2    ..........................................MVL   3
BraICE1-1   ..........................................MVL   3
BraICE1-2   ..........................................MAL   3
BcaICE1-1   ..........................................MVL   3
BcaICE1-2   .............MTRDGETTATKKAKKVARNLNCEKALTFTMVL  32
BcaICE1-3   .......................MIRIQNPPHGFLVKALNFTMVL  22
BoICE1-1    ..........................................MVL   3
BoICE1-2    ..........................................MVL   3
BnICE1-1    .......MLIHMTYHITINDGNEEKAKKVTRNLNCEKALTVTMVL  38
BnICE1-2    ..........................................MVL   3
BnICE1-3    ..........................................MAL   3
BnICE1-4    ..........................................MVL   3
BnICE1-5    ..........................................MVL   3
BnICE1-6    ..........................................MAL   3
BniICE1-1   ..........................................MVL   3
BniICE1-2   ..........................................MVL   3
BniICE1-3   ..........................................MAL   3
BjuICE1-1   ..........................................MVL   3
BjuICE1-2   ..........................................MVL   3
BjuICE1-3   MKSYWLVNLEVHLRGHSDKTKQRSHENSGNCLLCKIKFLTFAMAL  45
BjuICE1-4   ..........................................MVL   3
BjuICE1-5   ..........................................MVL   3
BjuICE1-6   ..........................................MAL   3
AtICE2      ............................................M   1
BrICE2-1    ............................................M   1
BrICE2-2    ............................................M   1
BraICE2-1   ..........................................MVL   3
BraICE2-2   ............................................M   1
BcaICE2-1   ............................................M   1
BcaICE2-2   ............................................M   1
BoICE2-1    ............................................M   1
BoICE2-2    ............................................M   1
BnICE2-1    ............................................M   1
BnICE2-2    ............................................M   1
BnICE2-3    ............................................M   1
BnICE2-4    ............................................M   1
BniICE2-1   ............................................M   1
BniICE2-2   ............................................M   1
BjuICE2-1   ............................................M   1
BjuICE2-2   ............................................M   1
BjuICE2-3   ............................................M   1
BjuICE2-4   ............................................M   1
Consensus
```

```
AtICE1       DGNNGGGVWLN......GGGG.........EREENEEGSWGRNQE   33
BrICE1-1     DGN.GGGVWLGGG.....GGGG.....GRVHEEE...........   61
BrICE1-2     DGN.GGGVWLG......GGGGGGGGGGERVQEEENEEASWGRNQE   41
BraICE1-1    DGN.GGGVWLGGG.....GGGG.....ERVHEEE...........   26
BraICE1-2    D.GNGGAVWLN......SSGE..........EEASWG...GNQEH   28
BcaICE1-1    DGN.GGAVWLGSGGGGGGGGGG.....ERVHEEE...........   31
BcaICE1-2    DGN.GGAVWLG...GGGGGGGG.....ERVHEEE...........   57
BcaICE1-3    DGN.RGGVWLN......GGGG...G..ERVQEEENEEASWGRNQE   55
BoICE1-1     DGN.GGAVWLG...GGGGGGGG.....ERVHEEE...........   28
BoICE1-2     DGN.GGGVWLG......GGGG..GG..ERVQEEENEEASWGRNQE   37
BnICE1-1     DGN.GGAVWLGGG.....GGGE.....S.VHGEE...........   60
BnICE1-2     DGN.GGGVWLG......GGGGGGGG..ERVQEEENEEASWGRNQE   39
BnICE1-3     D.GNGGAVWLN......SSGE..........EEASWG...GNQEH   28
BnICE1-4     DGN.GGAVWLG...GGGGGGG......ERVHEEE...........   27
BnICE1-5     DGN.GGGVWLG......GGGG..GG..ERVQEEENEEASWGRNQE   37
BnICE1-6     D.GNGGAVWLN......SSGE..........EEASWG...GNQEH   28
BniICE1-1    DGN.GGAVWLG....GGGGGRG.....ERVHEEEE..........   28
BniICE1-2    DGN.GGGVWLN......GGGG...G..ERAQEEENEEASWGRNQE   36
BniICE1-3    DDGNGGAVWLN......SSGEG....IQEENEEASWG...RNQED   35
BjuICE1-1    DGN.GGGVWLGGGS..GGGGGG.....ERVHEEE...........   29
BjuICE1-2    DGN.GGGVWLG......GGGGGGGE...RVQEEENEEASWGRNQE   38
BjuICE1-3    D.GNGGAVWLN......SSGE..........EEASWG...GNQEH   70
BjuICE1-4    DGN.GGGVWLN......GGGG...G..ERVQEEENEEASWGRNQE   36
BjuICE1-5    DGN.GGAVWLGSGGGGGGGGGG.....ERVHEEE...........   31
BjuICE1-6    DDGNGGAVWLN......SSGEG....IQEENEEASWG...RNQED   35
AtICE2       NSD...GVWLD......GSGES.........PEVN........NG   20
BrICE2-1     NGD...GVWLDD.....DAGER.........SQAG.......YND   22
BrICE2-2     NGN...GTWLH......DAGEP.........SQAN.......DND   21
BraICE2-1    DGN.GGGVWLG......GGGGGGGG..ERVQEEENEEASWGRNQE   39
BraICE2-2    NGN...GTWLH......DAGEP.........SQAN.......DND   21
BcaICE2-1    NGD...GTWLH......DAGQQ.........SQAGN......DND   22
BcaICE2-2    NGD...GVWLN......DPGER.........SLAS.......DND   21
BoICE2-1     NGD...GVWLD......DAGER.........SQAG.......YND   21
BoICE2-2     NGD...GTWLH......DAGEQ.........SQAGN......DND   22
BnICE2-1     NGD...GVWLDH.....DAGER.........SQAG.......YND   22
BnICE2-2     NGN...GTWLH......DAGEP.........SQAN.......DND   21
BnICE2-3     NGD...GVWLD......DAGER.........SQAG.......YND   21
BnICE2-4     NGD...GTWLH......DAGEQ.........SQAGN......DND   22
BniICE2-1    NGD...GTWLH......DAGEP.........SQAG.......DND   21
BniICE2-2    NGD...GVWLN......NAGER.........SHAS.......DND   21
BjuICE2-1    NG.....VWLDD.....DAGEH.........SQAG.......YND   20
BjuICE2-2    NGN...GTWLH......DAGEP.........SQAN.......DND   21
BjuICE2-3    NGD...GTWLH......DAGEP.........SQAG.......DND   21
BjuICE2-4    NGD...GVWLN......DPGER.........SLAS.......DND   21
Consensus            wl         g
```

```
AtICE1      DGS....SQFKPMLEG.....DWFSSNQP.HPQDLQMLQNQ.P.D    66
BrICE1-1    DGA....AQFKPMLEG....GDWFS.NQP...QELQSHH...Q.D    90
BrICE1-2    DGG.....QFKPMLEG...GGDWFTSNQP.HPQDLQMLQSQ.Q.D    75
BraICE1-1   DGA....AQFKPMLEG....GDWFS.NQP...QELQSHH...Q.D    55
BraICE1-2   VGASSLSHFKAPMLEG.....DWFSN.....PQDLHVLQNQ.Q.D    61
BcaICE1-1   DGG....AHFKPMLEA....GDWFSSNQP...HPQDLLH...Q.D    61
BcaICE1-2   DGA....AHFKPMLEG....GDWFS.NQPQDLHMLQSHH...Q.D    89
BcaICE1-3   DGGGASLSQFKPMLEGGG.GGDWFSSNQP.HPQDLQMLQSQ.Q.D    96
BoICE1-1    DGA....AHFKPMLEG....GDWFS.NQPQELHMLQSHH...Q.D    60
BoICE1-2    DGA.....QYKPMLEG...GGDWFTSNQP.HPQDLQMLQSQ.Q.D    71
BnICE1-1    DGA....AHFKPMLEE....GDWFS.NQP...QELQSHH...Q.D    89
BnICE1-2    DGG.....QFKPMLEG...GGDWFTSSQP.HPQDLQMLQSQ.Q.D    73
BnICE1-3    VGASSLSHFKAPMLEG.....DWFSN.....PQDLHVLQNQ.Q.D    61
BnICE1-4    NGA....AHFKPMLEG....VDWFS.NQPQELHMLQSHH...Q.D    59
BnICE1-5    DGA.....QYKPMLED...GGDWFTSNQP.HPQDLQMLQSQ.Q.D    71
BnICE1-6    VGASSLSHFKAPMLES.....DWFSNQQQHPQDLHVLQNQ.QED    67
BniICE1-1   DSG....AHFKPMLEA....GDWFSSNQP...HPQDLLH...Q.D    58
BniICE1-2   DGGGASLSQFKPMLEGGGGGGDWFSSNQP.HPQDLQMLQSQ.Q.D    78
BniICE1-3   GGASSLSHSRAPMLE.................DLHMLQNQ.Q.D    60
BjuICE1-1   DGA....AQFKPMLEE....GDWFS.NQP...QELQSHH...Q.D    58
BjuICE1-2   DGG.....QFKPMLEG...GGDWFTSSQP.HPQDLQMLQSQ.Q.D    72
BjuICE1-3   VGASSLSHFKAPMLEG.....DWFSN.....PQDLHVLQNQ.Q.D   103
BjuICE1-4   DGGGASLSQFKPMLE....GGDWFGSNQP.HPQDLQMLQSQ.Q.D    74
BjuICE1-5   DGG....AHFKPMLEA....GDWFSSNQP...HPQDLLH...Q.D    61
BjuICE1-6   GGASSLSHSRAPMLE.................DLHMLQNQ.Q.D    60
AtICE2      EAA....SWVRNPDE......DWFNNPPPPQ.......HTN.QND    47
BrICE2-1    EPV....PWVRNPEE......NWFNNSQP.........HDDSQNE    48
BrICE2-2    ESR....TWVRNTEE......NWFSNPQPLN.....PLHNN.QND    50
BraICE2-1   DGG.....QFKPMLEG...GGDWFTSNQP.HPQDLQMLQSQ.Q.D    73
BraICE2-2   ESR....TWVRNTEE......NWFSNPQPLN.....PLHNN.QND    50
BcaICE2-1   ESG....SWVRNTEE......NWFNNPQPLHHGSQTILHNNNQND    57
BcaICE2-2   EPV....PWVRNPEE......NWFNNMQPPH......PHDDSQNE    50
BoICE2-1    EPV....PWVRNPEE......NWFNNSQP.........HDDSQNE    47
BoICE2-2    ESG....SWVRNTEE......NWFNNPQPLHHGSQTILHNNNQND    57
BnICE2-1    ESV....PWVRNPEE......NWFNNTQP.........QDDSQNE    48
BnICE2-2    ESR....TWVRNTEE......NWFSNPQPLN.....PLHNN.QND    50
BnICE2-3    EPV....PWVRNPEE......NWFNNSQP.........HDDSQNE    47
BnICE2-4    ESG....SWVRNTEE......NWFNNPQPLHHGSQTILHNNNQND    57
BniICE2-1   EPG....SWVRNTEE......NWFSNPQPLHHGSQAILHN....D    52
BniICE2-2   EPV....PWVRNPEE......NWFNNMQPPN......PHGDSQNE    50
BjuICE2-1   EPV....PWVRNPEE......NWFNNSQP.........HDDSQNE    46
BjuICE2-2   ESR....TWVRNTEE......NWFSNPQPLN.....PLHNN.QND    50
BjuICE2-3   EPG....SWVRNTEE......NWFNNPQPLHHGAQ.VLHN....D    51
BjuICE2-4   EPV....PWVRNPEE......NWFNNMQPPH......PHDDSQNE    50
Consensus                 e
```

```
AtICE1      FRYFGG..FPFNPNDN.LLLQHS..IDSSSSCSP.SQAFSLDPS.    104
BrICE1-1    FRFLGG..FAFNPNDN...........LLLDSSS.SQAFGLDMS.    120
BrICE1-2    FRFLGG..FGFNPNDNLLLLQHS..MDSSSSCSP.SQAFSLDPS.    114
BraICE1-1   FRFLGG..FAFNPNDN...........LLLDSSS.SQAFGLDMS.     85
BraICE1-2   FRFLGG..FPFNPNDN.....LL..LDSSS.....SQPFTLDTS.     91
BcaICE1-1   FRFLGGGGFGFNPNDN......L..IDSSSSCSP.SQAFGLDMSH     97
BcaICE1-2   FRFLGGG.FAFNPNDN.....LL..LDSSSSCSP.SQAFGLDMS.    124
BcaICE1-3   FRFLGG..FGFNPNENLLLLQQS..MDSSSSCSP.SQAFSLDPS.    135
BoICE1-1    FRFLGGG.FAFNPNDN.....LL..LDSSSSCSP.SQAFGLDMS.     95
BoICE1-2    FRFLGG..FGFNPNDNLLLLHQS..MDSSSSCSP.SQAFSLDPS.    110
BnICE1-1    FRFLGG..FAFNPNDN...........LLLDSSS.SQAFGLDMS.    119
BnICE1-2    FRFLGG..FGFNPNDNLLLLQHS..MDSSSSCSP.SQAFSLDPS.    112
BnICE1-3    FRFLGG..FPFNPNDN.....LL..LDSSS.....SQPFTLDTS.     91
BnICE1-4    FRFLGGG.FAFHQS...........VDSSSSFSP.SQAFGLDMS.     90
BnICE1-5    FRFLGG..FGFNPNDNLLLLHQS..MDSSSSCSP.SQAFSLDPS.    110
BnICE1-6    FRFLGD..FLINPNDN.....LL..LDSSS.....SQLFTLDTS.     97
BniICE1-1   FRFLGGGGFGFNPNDN......L..IDSSSSCSP.SQAFGLDMSH     94
BniICE1-2   FRFLGG..FGFNPNENLLLLQQS..MDSSSSCSP.SQAFSLDPS.    117
BniICE1-3   FRFLGG..FAFNPNDN.....LL..LDSSSSCSP.SRVFSLDPS.     94
BjuICE1-1   FRFLGGGGCAFNPIDS...........YSS.CSP.SQAFSLDMS.     89
BjuICE1-2   FRFLGG..FGFNPNDNLLLLQHS..MDSSSSCSP.SQAFSLDPS.    111
BjuICE1-3   FRFLGG..FPFNPNDN.....LL..LDSSS.....SQPFTLDTS.    133
BjuICE1-4   FRFLGG..FGFNPNENLLLLQQS..MDSSSSCSP.SQAFSLDPS.    113
BjuICE1-5   FRFLGGGGFGFNPNDN......L..IDSSSSCSP.SQAFGLDMSH     97
BjuICE1-6   FRFLGG..FAFNPNDN.....LL..LDSSSSCSP.SRVFSLDPS.     94
AtICE2      FRFN.GG.FPLNPSENLLLLLQQ.SIDSSSSSSPLLHPFTLDAAS     89
BrICE2-1    FRFNNGG.FPLNPSENLLLLLQQ.SIDSSSP....LQHFTLDATT     87
BrICE2-2    FRFNSGA.FPSNPSENLLLLLQQ......................     72
BraICE2-1   FRFLGG..FGFNPNDNLLLLQHS..MDSSSSCSP.SQAFSLDPS.    112
BraICE2-2   FRFNSGA.FPSNPSENLLLLLQQ......................     72
BcaICE2-1   FRFS.GG.FPSNPSENLLLLLQQ.STDTSSQ....LQHFTLDP.T     94
BcaICE2-2   FRFNSGG.FPLNPSENLLFLLQQ.SIDSSSP....LQHLTLDSDT     89
BoICE2-1    FRFNNGG.FPLNPSENLLLLLQQ.SIDSSSP....FQHFTLDSAT     86
BoICE2-2    FRFS.GG.FPSNPSENLLLLLQQ.STDTSSQ....LQHFTLDP.T     94
BnICE2-1    FRFNNGG.FPLNPSENLLLLLQQ.SIDSSSP....FQHYTLDATT     87
BnICE2-2    FRFNSGA.FPSNPSENLLLLLQQ......................     72
BnICE2-3    FRFNNGG.FPLNPSENLLLLLQQ.SIDSSSP....FQHFTLDSAT     86
BnICE2-4    FRFS.GG.FPSNPSENLLLLLQQ.STDTSSQ....LQHFTLDP.T     94
BniICE2-1   LRFSTGG.LPLNPSENLLLLLQQQSIDTSSP....LQHFTLDP.A     91
BniICE2-2   FRFNSGG.FPLNPSENLLLLLQQ.SIDSSSP....LQPFTLDS.T     88
BjuICE2-1   FRFNNGG.FPLNPSENLLLLLQQ.SIDSSSP....FQHFTLDATT     85
BjuICE2-2   FRFNSGA.FPSNPSENLLLLLQQ......................     72
BjuICE2-3   LRFS.GG.LPLNPSENLLLLLQQQSIDTSSP....LQHFTLDP.A     89
BjuICE2-4   FRFNSGG.FPLNPSENLLFLLQQ.SIDSSSP....LQHLTLDSDT     89
Consensus    r
```

leucine-rich region

DNAMAN File: Multiple Alignment

```
AtICE1       QQNQFLST......NNNKGC....LLN.VPSSANP.....FDNAF     133
BrICE1-1     HQPSFLAA......ADNKSC....LLN.VPSSANP.....FDNAF     149
BrICE1-2     .QASFLAA......ANNKSC....LLNVVPSSANP.....FDNAF     143
BraICE1-1    HQPSFLAA......ADNKSC....LLN.VPSSANP.....FDNAF     114
BraICE1-2    .QQPSFLP......SNNKSC....LLS.APSDTNP.....FDNAF     119
BcaICE1-1    HHPSFLA........NNKSC....LLN....NPFD.....NN.AF     120
BcaICE1-2    HQPSFLAA......ANNKSC....LLN.V.ANPFD.....NN.AF     151
BcaICE1-3    SQASFLAA......ANNKSC....LLN.VPSSTNP.....FDNAF     164
BoICE1-1     HQPSFLAA......ANNKSC....LLN.V.ANPFD.....NN.AF     122
BoICE1-2     .QASFLAA......ANNKSC....LLN.VPSSANP.....FDNAF     138
BnICE1-1     HQPSFLAA......ADNKSC....LLN.VPSSANP.....FDNAF     148
BnICE1-2     .QASFLAA......ANNKSC....LLNVVPSSANP.....FDNAF     141
BnICE1-3     .QPSFFLP......SNNKSC....LLS.APSDTNP.....FDNAF     119
BnICE1-4     HQPSFLAA......ANNN.............NPFD.....NNNAF     111
BnICE1-5     .QASFLAA......ANNKSC....LLN.VPSSANP.....FDNAF     138
BnICE1-6     .QPS.FLP......SNNKSC....LLS.APSDTNP.....VDNAF     124
BniICE1-1    HQPSFLA........NSKSC....LLN....NPFD.....NN.AF     117
BniICE1-2    SQASFLAA......ANNKSC....LLN.VPSSANP.....FDNAF     146
BniICE1-3    .QPS.FLP......SNNKYC....LLN.VPSDTNP.....FDNAF     121
BjuICE1-1    HQLSFLAA......ANNNNH....FDN.N............NNAF     111
BjuICE1-2    .QASFLAA......ANNKSC....LLNVVPSSANP.....FDNAF     140
BjuICE1-3    .QPSFFLP......SNNKSC....LLS.APSDTNP.....FDNAF     161
BjuICE1-4    SQASFLAA......ANNKSC....LLN.VPSSANP.....FDNAF     142
BjuICE1-5    HHPSFLA........NNKSC....LLN....NPFD.....NN.AF     120
BjuICE1-6    .QPS.FLP......SNNKYC....LLN.VPSDTNP.....FDNAF     121
AtICE2       QQQQQQQQQQEQSFLATKAC.IVSLLN.VPTINNN.......TFD     125
BrICE2-1     PQQQQQQQQ...SFLATKAC.IASLLT.VPTTTNN..NNNN.PFD     124
BrICE2-2     EQQQQQQ.....SFLTTKAC.MASLLN.IPTSNTN.NNINSNPFD     109
BraICE2-1    .QASFLAA......ANNKSC....LLNVVPSSANP.....FDNAF     141
BraICE2-2    EQQQQQQQ....SFLTTKAC.MASLLN.IPTSNTN.NNINSNPFD     110
BcaICE2-1    PQEQQQQQQ.EQSFLATKAC.ITSLLN.VPTSNSNTNNINNNPFD     136
BcaICE2-2    PQQQQQQQ....SFFATKAC.IASLLN.LPPNN.........TFD     119
BoICE2-1     PQQQQQQQ....YFLATKAC.IASLLN.VPTTTNN....NN.PFD     120
BoICE2-2     PQEQQQQS.....FLATKAC.ITSLLN.VPTSNN......NNPFD     126
BnICE2-1     PQQQQQQQQQ...SFLATKAC.IASLLT.VPTTTNN..NNNNNPFD    125
BnICE2-2     EQQQQQQQ....SFLTTKAC.MASLLN.IPTSNTN.NNINSNPFD     110
BnICE2-3     PQQQQQQQ....YFLATKAC.IASLLN.VPTTNNN....NN.PFD     120
BnICE2-4     PQEQQQQS.....FLATKAC.ITSLLN.VPTSNN......NNPFD     126
BniICE2-1    PQQQQQQQ..EQSFLATKACGITSLLN.VPTSNN.....NNNPFD     128
BniICE2-2    PQQQQ..H....SFLATKAC.IASLLN.VPPNNNN.......PFD     118
BjuICE2-1    PQQQQQQQQ...SVLATKAC.IASLLT.VPTTTNN..NNNN.PFD     122
BjuICE2-2    EQQQQQQ.....SFLTTKAC.MASLLN.IPTSNTN.NNINSNPFD     109
BjuICE2-3    PQQQQQQQQQEQSFLATKACGITSLLN.VPTSNN.....NINPFD     128
BjuICE2-4    PQQQQQQQ....SFFATKAC.IASLLN.LPPNN.........TFD     119
Consensus
```

glutamine-rich region

```
AtICE1       EFGSES.GFLNQI.....HAPISMGFGSLTQLGNRDLSSVPDFLS    172
BrICE1-1     EFGSDS.GFLGHI.....QAP..MGFGSLMQLS............    174
BrICE1-2     EFGSDS.GFLNQI.....QAPVSMGFGSLTQLG....SSVPDFLS    178
BraICE1-1    EFGSDS.GFLGHI.....QAP..MGFGSLMQLS............    139
BraICE1-2    EFGSDS.CFLG................PLTQ..............    133
BcaICE1-1    EFGSDS.GFLGHI.....QPP..MGFGSLMQLS............    145
BcaICE1-2    EFGSDS.GFLGHI.....QAP..MGFGSLMQLGN...........    177
BcaICE1-3    EFGSDS.GFLSQI.....QAPVSMGFGSLTQLG....SSVPDFLS    199
BoICE1-1     EFGSDS.GFLGHI.....QAP..MGFGSLMQLGN...........    148
BoICE1-2     EFGSDS.GFLNQI.....QAPVSMGFGSLTQLG....SSVPDFLS    173
BnICE1-1     EFGSDS.GFLGHI.....QAP..MGFGSLMQLS............    173
BnICE1-2     EFGSDS.GFLNQI.....QAPVSMGFGSLTQLG....SSVPDFLS    176
BnICE1-3     EFGSDS.CFLG................PLTQ..............    133
BnICE1-4     EFGSDS.GFLGHI.....QAP..MGFGSLMQLGN...........    137
BnICE1-5     EFGSDS.SFLNQI.....QAPVSMGFGSLTQLG....SSVPDFLS    173
BnICE1-6     ELGSDS.GFLGQ............AFGSLTQ..............    142
BniICE1-1    EFGSDS.GFLGHI.....QPP..MGFGSLMQLS............    142
BniICE1-2    EFGSDS.GFLSQI.....QAPVSMGFGSLTQLG....SSVPDFLS    181
BniICE1-3    EFGSDS.GFLGQ............GFGSLTNREM...TSVPDFLS    150
BjuICE1-1    EFGSDS.GFLGHI.....QAP..MGFGSLMQLS............    136
BjuICE1-2    EFGSDS.GFLNQI.....QAPVSMGFGSLTQLG....SSVPDFLS    175
BjuICE1-3    EFGSDS.CFLG................PLTQ..............    175
BjuICE1-4    EFGSDS.GFLSQI.....QAPVSMGFGSLTQLG....SSVPDFLS    177
BjuICE1-5    EFGSDS.GFLGHI.....QPP..MGFGSLMQLS............    145
BjuICE1-6    EFGSDS.GFLGQ............GFGSLTNREM...TSVPDFLS    150
AtICE2       DFGFDS.GFLGQQFHGNHQSPNSMNFTGLNHSV............    157
BrICE2-1     DFGFDS.PFLGQLN....QTPNSMSFPVMTSPT............    152
BrICE2-2     ELGFSS.GFLGQT....NQTPLSMSFSGMSSP.............    136
BraICE2-1    EFGSDS.GFLNQI.....QAPVSMGFGSLTQLG....SSVPDFLS    176
BraICE2-2    ELGFSS.GFLGQT....NQTPLSMSFSGMSSP.............    137
BcaICE2-1    ELGFSS.GFSG.................MSSP.............    150
BcaICE2-2    DFGFDS.AFLGQL.....QTPNSMSFPVMTSP.............    145
BoICE2-1     DFGFDS.PFLGQLN....QTPNSMSFPVMTSPP............    148
BoICE2-2     ELGFSS.GFFGQTHV..NQTPNSMSFAGMSSP.............    155
BnICE2-1     DFGFNS.PFLGQLN....QTQNSMSFPVMTSPT............    153
BnICE2-2     ELGFSS.GFLGQT....NQTPISMSFSGMSSP.............    137
BnICE2-3     DFGFDS.PFLGQLN....QTPNSMSFPVMTSPP............    148
BnICE2-4     ELGFSS.GFFGQTHV..NQTPNSMSFAGMSSP.............    155
BniICE2-1    ELGFGS.GFFGQTHV..NQTPNSMNFAGMSSP.............    157
BniICE2-2    DFGFDSPLFLGQL.....QTPNSMSLPVMTSP.............    145
BjuICE2-1    NFGFDS.PFLGQLN....QTPNSMSFPVMTSPP............    150
BjuICE2-2    ELGFSS.GFLGQP....NQTPISMSFSGMSSP.............    136
BjuICE2-3    ELGFSS.GFFGQAHV..NQTPNSMNFAVMSSP.............    157
BjuICE2-4    DFGFDS.AFLGQL.....QTPNSMSFPVMTSP.............    145
Consensus      g  s  f
```

```
AtICE1       ARSLLAPESNNNNTMLCGGFT..........APLELEGFG.SPAN    206
BrICE1-1     ...........SVPELCGGGGGGG.....GFTPLESEGFG.SLAS    202
BrICE1-2     ARSLLPP.ENNNTAPLCGGGGGGG.....GFTALELEGFG.SPAS    216
BraICE1-1    ...........SVPELCGGGG........GFTPLETEGFG.SLAS    164
BraICE1-2    .............................GFTPLELQGFT.SPAK    148
BcaICE1-1    ...........SVPELGSGGG.........FTPLELEGFG.SPAS    169
BcaICE1-2    .........NNSTPLCGGGGGGE.......FTPLELEGFG.SPAS    205
BcaICE1-3    SRSLLQ..ENNNTTSLCGGGGGGGGGGGGGFTPLELEGFG.SPAS    241
BoICE1-1     .........NNSTPLCGGGGGGGG.....GFTPLELEGFG.SPAS    178
BoICE1-2     ARSLLPP.ENNNTTPLCGGGGGGGGGGGGGFTPLELEGFG.SPAS    216
BnICE1-1     ...........SVPELCGGGGGGG.....GLTPLESEGFG.SLAS    201
BnICE1-2     ARSLLPP.ENNNTAPLCGGGGS.......GFTPLELEGFG.SPAS    212
BnICE1-3     .............................GFTPLELQGFT.SPAK    148
BnICE1-4     .........NNSTPLCGGGG..........FTPLELEGFG.SPAS    162
BnICE1-5     ARSLLPP.ENNNTPPLCGGGGGGGGGGG..FTPLELEGFG.SPAS    214
BnICE1-6     .............................GFTPLELQGFT.SPAK    157
BniICE1-1    ...........SVPELGSGGG.........FTPLELEGFG.SPAS    166
BniICE1-2    SRSLLQ..ENNNTTPLCGGGGGG.....GGFTPLELEGFG.SPAS    218
BniICE1-3    APV......NNNSTMLYGGGGEG......GFTPLELQGFITSPAK    183
BjuICE1-1    ...........SVPELCGGGG........GLTLLESEGFG.SLAS    161
BjuICE1-2    ARSLLPP.ENNNTAPLCGGGGGGG.....GFTALELEGFG.SPAS    213
BjuICE1-3    .............................GFTPLELQGFT.SPAK    190
BjuICE1-4    SRSLLQ..ENNNTTSLCGGGGGG....GGGFTPLELEGFG.SPAS    215
BjuICE1-5    ...........SVPELGSGGG.........FTPLELEGFG.SPAS    169
BjuICE1-6    APV......NNNSTMLYGGGGEG......GFTPLELQGFITSPAK    183
AtICE2       .............PDFLPAP......ENSSGS....CG.....LS    174
BrICE2-1     .............PDFLSSR......SCNVFSPLEFEG.L...AS    174
BrICE2-2     .............PDFLSSRPIPPP.ENSSFTPLEFAG.V...AN    163
BraICE2-1    ARSLLPP.ENNNATPLCGGGGG.......GFTPLELEGFG.SPAS    212
BraICE2-2    .............PDFLSSRSIPPP.ENSSFTPLEFAG.V...AN    164
BcaICE2-1    .............PENTAG.......VSNRFSPLEFAG.V...AN    171
BcaICE2-2    .............PDFLSSK......SCNVFSPLEFEGVV...AS    168
BoICE2-1     .............PDFLSSR......SCNVFSPLEFEG.V...AS    170
BoICE2-2     .............PENTAG.......VSNRFSPLEFTG.V...AN    176
BnICE2-1     .............PEFLSSR......SCNVFSPLEFEG.L...AS    175
BnICE2-2     .............PDFLSSRSIPPP.ENSSFTPLEFAG.V...AN    164
BnICE2-3     .............PDFLSSR......SCNVFSPLEFEG.V...AS    170
BnICE2-4     .............PENTAG.......VSNRFSPLEFTG.V...AN    176
BniICE2-1    .............PGNTTG.......VGNRFSPLEFAG.V...AN    178
BniICE2-2    .............PDFFSSK......SCNVFSPLEFEGVV...AS    168
BjuICE2-1    .............PDFLPSR......SCNVFSPLEFEG.L...AS    172
BjuICE2-2    .............PDFLSSRSIPPP.ENSSFTPLEFAG.V...AN    163
BjuICE2-3    .............PENTTG.......VGNRFSPLEFAG.V...AN    178
BjuICE2-4    .............PDFLSSK......SCNVFSPLEFEGVV...AS    168
Consensus                                         g
```

```
AtICE1      GGFVGNR.AKVLKPLEVLASSG..AQPTLFQKRAAMRQSSGSKMG   248
BrICE1-1    GGFVGSR.AKVLKPLEVLASSG..AQPTLFQKRAAMRQSSG....   240
BrICE1-2    ..FVGSR.PKVLKPLEVLASSG..AQPTLFQKRAAMRQSSGSKMG   256
BraICE1-1   GGFVGSR.AKVLKPLEVLASSG..AQPTLFQKRAAMRQSSG....   202
BraICE1-2   ..........VLKPLEVLASSSSGGQPTLFQKRAAMRQSSGSKFG   183
BcaICE1-1   ..FVGNRAAKVLKPLEVLASSG..AQPTLFQKRAAMRQSSGM...   207
BcaICE1-2   ..FVGSR.AKVLKPLEVLASSG..AQPTLFQKRAAMRQSSGM...   242
BcaICE1-3   ..FVGNR.AKVLKPLEVLASSG..AQPTLFQKRAAMRQSSGSKMG   281
BoICE1-1    ..FVGSR.AKVLKPLEVLASSG..AQPTLFQKRAAMRQSSGM...   215
BoICE1-2    ..FVGSR.PKVLKPLEVLASSG..AQPTLFQKRAAMRQSSGSKMG   256
BnICE1-1    GGFVGSR.AKVLKPLEVLASSG..AQPTLFQKRAAMRQSSG....   239
BnICE1-2    ..FVGSR.PKVLKPLEVLASSG..AQPTLFQKRAAMRQSSGSKMG   252
BnICE1-3    ..........VLKPLEVLASSS.GGQPTLFQKRAAMRQSSETKFG   182
BnICE1-4    ..FVGSR.AKVLKPLEVLASSG..AQPTLFQKRAAMRQSSGM...   199
BnICE1-5    ..FVGSR.PKVLKPLEVLASSG..AQPTLFQKRAAMRQSSGSKMG   254
BnICE1-6    ..........VLKPLEVLASSS.GGQPTLFQKRAAMRQSSETKFG   191
BniICE1-1   ..FVGNRSAKVLKPLEVLASSG..AQPTLFQKRAAMRQSSGR...   204
BniICE1-2   ..FVGNR.AKVLKPLEVLASSG..AQPTLFQKRAAMRQSSGSKMG   258
BniICE1-3   ..........VLKPLEVLASSG..AQPTLFQKRAAMRQSSGSKFG   216
BjuICE1-1   GGFVGSR.AKVLKPLEVLASSG..AQPTLFQKRAAMRQSSG....   199
BjuICE1-2   ..FVGSR.PKVLKPLEVLASSG..AQPTLFQKRAAMRQSSGSKMG   253
BjuICE1-3   ..........VLKPLEVLASSS.GGQPTLFQKRAAMRQSSETKFG   224
BjuICE1-4   ..FVGNR.AKVLKPLEVLASSG..AQPTLFQKRAAMRQSSGSKMG   255
BjuICE1-5   ..FVGNRAAKVLKPLEVLASSG..AQPTLFQKRAAMRQSSGM...   207
BjuICE1-6   ..........VLKPLEVLASSG..AQPTLFQKRAAMRQSSGSKFG   216
AtICE2      PLFSNR..AKVLKPLQVMASSG..SQPTLFQKRAAMRQSSSSK..   213
BrICE2-1    GVFENR..VKVLKPLEVLASSG..SEPTLFQKRAAVRESSSSK..   213
BrICE2-2    GVFENR..AKVLKPLDALASST..SQPTLFQKRAAMRQSSSSK..   202
BraICE2-1   ..FVGSR.PKVLKPLE.............................   225
BraICE2-2   GVFENR..AKVLKPLEVLASST..SQPTLFQKRAAMRQSSSSK..   203
BcaICE2-1   GVFENR..AKVLKPLDVLASST..SQPTLFQKRAAMRQSSSSK..   210
BcaICE2-2   GVFENR..AKVLKPLEFSPSSG..SQPTLFQKRAAMRESSVSK..   207
BoICE2-1    GVFENR..AKVLKPLEVLPSSG..SEPTLFQKRAAMHQSWSSK..   209
BoICE2-2    GVFENR..AKVLKPLDVLASST..SQPTLFQKRAAMRQSSSSK..   215
BnICE2-1    GVYENR..AKVLKPLEVLPSSG..SELTLFQKRAAMRESSSSK..   214
BnICE2-2    GVFENR..AKVLKPLEVLASST..SQPTLFQKRAAMRQSSSSK..   203
BnICE2-3    GVFENR..AKVLKPLEVLPSSG..SEPTLFQKRAAMHQSWSSK..   209
BnICE2-4    GVFENR..AKVLKPLDVLASST..SQPTLFQKRAAMRQSSSSK..   215
BniICE2-1   GVFENR..AKVLKPLDVLASSS..SQPTLFQKRAAMRQSSSSK..   217
BniICE2-2   GVFENR..AKVLKPLEVSPSSG..SQPTLFQKRAAMRESSVSK..   207
BjuICE2-1   GVFENR..AKVLKPLEVLPSSG..SELTLFQKRAAMRESSSSK..   211
BjuICE2-2   GVFENR..AKVLKPLEVLASST..SQPTLFQKRAAMRQSSSSK..   202
BjuICE2-3   GVFENR..AKVLKPLDVLASSS..SQPTLFQKRAAMRQSSSSK..   217
BjuICE2-4   GVFENR..AKVLKPLEFSPSSG..SQPTLFQKRAAMRESSVSK..   207
Consensus             vlkpl
```

AtICE1	NSESSGMRRFSDDGDMDETGIEVSGLNYESDEINESG.KAAESVQ	292
BrICE1-1	......RRKLSDDGEVDETGVEVSGLNYESDELNESG.KAAESVQ	278
BrICE1-2	NSESSGMRRLSDDGDMDETGVEVSGLNYESDELNESG.KASESVQ	300
BraICE1-1	......RRKLSDDGEVDETGVEVSGLNYESDELNESG.KAAESVQ	240
BraICE1-2	NS....ERKLSDETGLHYE..........SDEMNQSG.RAAESVE	213
BcaICE1-1	......MRKLSGDGEMDETGVEVSGLNYESDELNESGGKVSESVQ	246
BcaICE1-2	......MRKLSDDGEMDETGVEVSGLNYESDELNESG.KAAESVQ	280
BcaICE1-3	NSESSGMRRMSDDCDMDETGIEVSGLNYESDELNENG.KASESVQ	325
BoICE1-1	......MRKLSDDGEMDETGVEVSGLNYESDELNESG.KAAESVQ	253
BoICE1-2	NSESSGMRGLSDDGDMDETGVEVSGLNYESDELNESG.KASESVQ	300
BnICE1-1	......RRKLSDDGEVDETGVDVSGLNYESDELNESG.KAAESVQ	277
BnICE1-2	NSESSGMRRLSDDGDMDETGVEVSGLNYESDELNESG.KASESVQ	296
BnICE1-3	NS....ERKLSDDG..........................EIESVQ	198
BnICE1-4	......MRKLSDDGEMDETGVEVSGLNYESDELNESG.KAAESVQ	237
BnICE1-5	NSESSGMRRLSDDGDMDETGVEVSGLNYESDELNESG.KASESVQ	298
BnICE1-6	NS....ETKLSDDG..........................EIESVQ	207
BniICE1-1	......MRKLSDDGEMDETGVEVSGLNYESDELNESGKAASESVQ	243
BniICE1-2	NSESSGMRRMSDDCDMDETGIEVSGLNYESDELNENG.KASESVQ	302
BniICE1-3	NS....ERKLSDDGEMDETGIEISGLLYESDELNQRG.KAAESVE	256
BjuICE1-1	......RRKLSDDGEVDETGVEVSGLNYESDELNESG.KAAESVQ	237
BjuICE1-2	NSESSGMRRLSDDGDMDETGVEVSGLNYESDELNESG.KASESVQ	297
BjuICE1-3	NS....ERKLSDDG..........................EIESVQ	240
BjuICE1-4	NSESSGMRRMSDDCDMDETGIEVSGLNYESDELNENG.KASESVQ	299
BjuICE1-5	......MRKLSGDGEMDETGVEVSGLNYESDELNESGGKVSESVQ	246
BjuICE1-6	NS....ERKLSDDGEMDETGIEISGLLYESDELNQRG.KAAESVE	256
AtICE2	.....MCNSESSS.EMRKSSYER.EIDDTSTGIIDISGLNYESDD	251
BrICE2-1	.....MCNSDSSSSEVRR.....EVGDETSRGIVDI..FNYESDE	246
BrICE2-2	.....TCNSESSS.EMRRSSYEP.DIDDTSTG...I..ID.ISDE	234
BraICE2-1	....	225
BraICE2-2	.....TCNSESSS.EMRRSSYEP.DIDDASTG...I..IDIISDE	236
BcaICE2-1	.....TCNSESSS.EMRKSSYERENLDDASTG...I..IDIISDE	244
BcaICE2-2	.....MCNSESSSSEMRRSSYEREVVDDTSVG......FNYESDE	241
BoICE2-1	.....MCNSDSSSSEMRR.....EVGDETSTGIVDI..FNYESDE	242
BoICE2-2	.....TCNSESSS.EMRRSSYEP.DIDDASTG...I..IDIISDE	248
BnICE2-1	.....MCNSDSSSSEVRR.....EVGDETSPGIVDI..FNYESDE	247
BnICE2-2	.....TCNSESSS.EMRRSSYEP.DIDDASTG...I..IDIISDE	236
BnICE2-3	.....MCNSDSSSSEMRR.....EVGDETSTGIVDI..FNYESDE	242
BnICE2-4	.....TCNSESSS.EMRRSSYEP.DIDDASTG...I..IDIISDE	248
BniICE2-1	.....TCNSESSS.EMRRSSYERENLDDASTG...I..IDIISDE	251
BniICE2-2	.....MCNSESSSSEMKRSSYEREVVDDTSIG......FNYESDE	241
BjuICE2-1	.....MCNSDSSSSEVRR.....EVGDETSRGIVDI..FNYESDE	244
BjuICE2-2	.....TCNSESSS.EMRRSSYEP.DIDDASTG...I..IDIISDE	235
BjuICE2-3	.....TCNSESSS.EMRRSSYERENLDDASTG...I..IDIISDE	251
BjuICE2-4	.....MCNSESSSSEMRRSSYEREVVDDTSVG......FNYESDE	241
Consensus		

```
AtICE1       IGGGG.KGKKK.GMPAKNLMAERRRRKKLNDRLYMLRSVVPKIS.    334
BrICE1-1     NGGGG.RGKKK.GMPAKNLMAERRRRKKLNDRLYMLRSVVPKIS.    320
BrICE1-2     NGGG..KGKKK.GMPAKNLMAERRRRKKLNDRLYMLRSVVPKIS.    341
BraICE1-1    NGGGG.RGKKK.GMPAKNLMAERRRRKKLNDRLYMLRSVVPKIS.    282
BraICE1-2    NIRGS.KGKKK.GLPAKNLMAERRRRKKLNDRLYMLRSIVPKIS.    255
BcaICE1-1    NGGGG.KGKKK.GMPAKNLMAERRRRKKLNDRLYMLRSVVPKIS.    288
BcaICE1-2    NGGGG.RGKKK.GMPAKNLMAERRRRKKLNDRLYMLRSVVPKIS.    322
BcaICE1-3    NGGGG.KGKKK.GMPAKNLMAERRRRKKLNDRLYMLRSVVPKIS.    367
BoICE1-1     NGGGG.RGKKK.GMPAKNLMAERRRRKKLNDRLYMLRSVVPKIS.    295
BoICE1-2     NGGG..KGKKK.GMPAKNLMAERRRRKKLNDRLYMLRSVVPKIS.    341
BnICE1-1     NGGGG.RGKKK.GMPAKNLMAERRRRKKLNDRLYMLRSVVPKIS.    319
BnICE1-2     NGGG..KGKKK.GMPAKNLMAERRRRKKLNDRLYMLRSVVPKIS.    337
BnICE1-3     .SRGG.KDKKK.GLPAKNLMAERRRRKKLNDRLYMLRSIVPKIS.    239
BnICE1-4     NGGGG.RGKKK.GMPAKNLMAERRRRKKLNDRLYMLRSVVPKIS.    279
BnICE1-5     NGGG..KGKKK.GMPAKNLMAERRRRKKLNDRLYMLRSVVPKIS.    339
BnICE1-6     .SRGG.KGKKK.GLPAKNLMAERRRRKKLNDRLYMLRSIVPKIS.    248
BniICE1-1    NGGGG.KGKKK.GMPAKNLMAERRRRKKLNDRLYMLRSVVPKIS.    285
BniICE1-2    NGGG..KGKKK.GMPAKNLMAERRRRKKLNDRLYMLRSVVPKIS.    343
BniICE1-3    .NRGG.KGKKKKGIPAKNLMAERRRRKKLNDRLYMLRSVVPKIS.    298
BjuICE1-1    NGGGG.RGKKK.GMPAKNLMAERRRRKKLNDRLYMLRSVVPKIS.    279
BjuICE1-2    NGGG..KGKKK.GMPAKNLMAERRRRKKLNDRLYMLRSVVPKIS.    338
BjuICE1-3    .SRGG.KDKKK.GLPAKNLMAEKRRRKKLNDRLYMLRSIVPKIS.    281
BjuICE1-4    NGGG..KGKKK.GMPAKNLMAERRRRKKLNDRLYMLRSVVPKIS.    340
BjuICE1-5    NGGGG.KGKKK.GMPAKNLMAERRRRKKLNDRLYMLRSVVPKIS.    288
BjuICE1-6    .NRGG.KGKKKKGIPAKNLMAERRRRKKLNDRLYMLRSVVPKIS.    298
AtICE2       HNTNNNKGKK.KGMPAKNLMAERRRRKKLNDRLYMLRSVVPKIS.    294
BrICE2-1     HNNN..KGKK.KGMPAKNLMAERRRRKKLNDRLYMLRSVVPKIS.    287
BrICE2-2     HN....KGKK.KGMPAKNLMAERRRRKKLNDRLYMLRSVVPKIS.    273
BraICE2-1    .............................................    225
BraICE2-2    HNN...KGKK.KGMPAKNLMAERRRRKKLNDRLYMLRSVVPKIS.    276
BcaICE2-1    HNNN..KGKK.KGLPAKNLMAERRRRKKLNDRLYMLRSVVPKIS.    285
BcaICE2-2    HNNK..KGKK.KGMPAKNLMAERRRRKKLNDRLYMLRSVVPNIS.    282
BoICE2-1     HNNN..KGKK.KGMPAKNLMAERRRRKKLNDRLYMLRSVVPKIS.    283
BoICE2-2     HN....KGKK.KGMPAKNLMAERRRRKKLNDRLYMLRSVVPKIS.    287
BnICE2-1     HNHN..KGKK.KGMPAKNLMAERRRRKKLNDRLYMLRSVVPKISK    289
BnICE2-2     HNN...KGKK.KGMPAKNLMAERRRRKKLNDRLYMLRSVVPKIS.    276
BnICE2-3     HNNN..KGKK.KGMPAKNLMAERRRRKKLNDRLYMLRSVVPKIS.    283
BnICE2-4     HN....KGKK.KGMPAKNLMAERRRRKKLNDRLYMLRSVVPKIS.    287
BniICE2-1    HNNN..KSKKNKGMPAKNLMAERRRRKKLNDRLYMLRSVVPKIS.    293
BniICE2-2    HHNN..KGKK.KGMPAKNLMAERRRRKKLNDRLYMLRSVVPNIS.    282
BjuICE2-1    HNNN..KGKK.KGMPAKNLMAERRRRKKLNDRLYMLRSVVPKIS.    285
BjuICE2-2    HNN...KGKK.KGMPAKNLMAERRRRKKLNDRLYMLRSVVPKIS.    275
BjuICE2-3    HNNN..KSKKNKGMPAKNLMAERRRRKKLNDRLYMLRSVVPKIS.    293
BjuICE2-4    HNNK..KGKK.KGMPAKNLMAERRRRKKLNDRLYMLRSVVPNIS.    282
Consensus
```

MYC-like bHLH domain

```
AtICE1      ........................KMDRASILGDAIDYLKELLQR   355
BrICE1-1    ........................KMDRASILGDAIDYLKELLQR   341
BrICE1-2    ........................KMDRASILGDAIDYLKELLQR   362
BraICE1-1   ........................KMDRASILGDAIDYLKELLQR   303
BraICE1-2   ........................KMDRASILGDAIDYLKELLQR   276
BcaICE1-1   ........................KMDRASILGDAIDYLKELLQR   309
BcaICE1-2   ........................KMDRASILGDAIDYLKELLQR   343
BcaICE1-3   ........................KMDRASILGDAIDYLKELLQR   388
BoICE1-1    ........................KMDRASILGDAIDYLKELLQR   316
BoICE1-2    ........................KMDRASILGDAIDYLKELLQR   362
BnICE1-1    ........................KMDRASILGDAIDYLKELLQR   340
BnICE1-2    ........................KMDRASILGDAIDYLKELLQR   358
BnICE1-3    ........................KMDRASILGDAIDYLKELLQR   260
BnICE1-4    ........................KMDRASILGDAIDYLKELLQR   300
BnICE1-5    ........................KMDRASILGDAIDYLKELLQR   360
BnICE1-6    ........................KMDRASILGDAIDYLKELLQR   269
BniICE1-1   ........................KMDRASILGDAIDYLKELLQR   306
BniICE1-2   ........................KMDRASILGDAIDYLKELLQR   364
BniICE1-3   ........................KMDRASILGDAIDYLKELLQR   319
BjuICE1-1   ........................KMDRASILGDAIDYLKELLQR   300
BjuICE1-2   ........................KMDRASILGDAIDYLKELLQR   359
BjuICE1-3   ........................KMDRASILGDAIDYLKELLQR   302
BjuICE1-4   ........................KMDRASILGDAIDYLKELLQR   361
BjuICE1-5   ........................KMDRASILGDAIDYLKELLQR   309
BjuICE1-6   ........................KMDRASILGDAIDYLKELLQR   319
AtICE2      ........................KMDRASILGDAIDYLKELLQR   315
BrICE2-1    ........................KMDRASILGDAIDYLKELLQR   308
BrICE2-2    ........................KMDRASILGDAIDYLKELLQR   294
BraICE2-1   .........................MDRASILGDAIDYLKELLQR   245
BraICE2-2   ........................KMDRASILGDAIDYLKELLQR   297
BcaICE2-1   ........................KMDRASILGDAIDYLKELLQR   306
BcaICE2-2   ........................KMDRASILGDAIDYLKELLQK   303
BoICE2-1    ........................KMDRASILGDAIDYLKELLQR   304
BoICE2-2    ........................KMDRASILGDAIDYLKELLQR   308
BnICE2-1    VSKDSYLLLFFFFFFVILNCGFYMQMDRASILGDAIDYLKELLQR   334
BnICE2-2    ........................KMDRASILGDAIDYLKELLQR   297
BnICE2-3    ........................KMDRASILGDAIDYLKELLQR   304
BnICE2-4    ........................KMDRASILGDAIDYLKELLQR   308
BniICE2-1   ........................KMDRASILGDAIDYLKELLQR   314
BniICE2-2   ........................KMDRASILGDAIDYLRELLQK   303
BjuICE2-1   ........................KMDRASILGDAIDYLKELLQR   306
BjuICE2-2   ........................KMDRASILGDAIDYLKELLQR   296
BjuICE2-3   ........................KMDRASILGDAIDYLKELLQR   314
BjuICE2-4   ........................KMDRASILGDAIDYLKELLQK   303
Consensus                            mdrasilgdaidyl ellq
```

```
AtICE1       INDLHNELES.TPPGS....LPPTSSSFHPLTPTPQTLSCRVKEE   395
BrICE1-1     INDLHNELES.TPNGS....LPLASSSFHPLTPTPQTLSCRVKEE   381
BrICE1-2     INDLHNELES.TPTGS....LPPTSSSFHPLTPTPQTLSCRVKEE   402
BraICE1-1    INDLHNELES.TPNGS....LPLASSSFHPLTPTPQTLSCRVKEE   343
BraICE1-2    INDLHNELDQSTPTPP..GSLPQTPSSFHPLTPTPQSLSCHVKEE   319
BcaICE1-1    INDLHNELES.TPNGSS...LPPTSSSLHPLTPTPQTLSCRVKEE   350
BcaICE1-2    INNLHNELES.TPNGS....LPPTSSSFHPLTPTPQTLSCRVKEE   383
BcaICE1-3    INDLHNELES.TPTGS....LPPTSSSFHPLTPTPQTLSCRVKEE   428
BoICE1-1     INDLHNELES.TPNGS....LPPTSSSFHPLTPTPQTLSCRVKEE   356
BoICE1-2     INDLHTELES.TPAGS....LPPTSSSFHPLTPTPQTLSCRVKEE   402
BnICE1-1     INDLHNELES.TPNGS....LPLASSSFHPLTPTPQTLSSRVKEE   380
BnICE1-2     INDLHNELES.TPTGS....LPPTSSSFHPLTPTPQTLSCRVKEE   398
BnICE1-3     INDLHNELES.TPTPP..GSLPQTP..........QSLSCHVKEE   292
BnICE1-4     INDLHNELES.TPNGS....LPPTSSSFHPLTPTPQTLSCRVKEE   340
BnICE1-5     INDLHNELES.TPAGS....LPPTSSSFHPLTPTPQTLSCRVKEE   400
BnICE1-6     INDLHDELES.TPTPPPPGSLPQTP..........QSLSCHVKEE   303
BniICE1-1    INDLHNELES.TPNGSS...LPPTSSSLHPLTPTPQTLSCRVKEE   347
BniICE1-2    INDLHNELES.TPTGS....LPPTSSSFHPLTPTPQTLSCRVKEE   404
BniICE1-3    INDLHNELEA.TPTPP..GSLPPTPSSFHPLTPTTQTLSFPVKEE   361
BjuICE1-1    INDLHNELES.TPNGS....LPLASSSFHPLTPTPQTLSCRVKEE   340
BjuICE1-2    INDLHNELES.TPTGS....LPPTSSSFHPLTPTPQTLSCRVKEE   399
BjuICE1-3    INDLHNELES.TPTPP..GSLPQTP..........QSLSCHVKEE   334
BjuICE1-4    INDLHNELES.TPTGS....LPPTSSSFHPLTPTPQTLSCRVKEE   401
BjuICE1-5    INDLHNELES.TPNGSS...LPPTSSSLHPLTPTPQTLSCRVKEE   350
BjuICE1-6    INDLHNELEA.TPTPP..GSLPPTPSSFHPLTPTTQTLSFPVKEE   361
AtICE2       INDLHTELES....T......PPSSSSLHPLTPTPQTLSYRVKEE   350
BrICE2-1     INDLHTELES....T......PSSSSGLTP...SPQTLPYRVKEE   340
BrICE2-2     INDLHTELES....TA.....PPSSSSLNPLTPTTQTLSYRVKEE   330
BraICE2-1    INDLHNELES.TPTGS....LPPTSSSFHPLTPTPQTLSCRVKEE   285
BraICE2-2    INDLHTELES....TA.....PPSSSSLNPLTPTTQTLSYRVKEE   333
BcaICE2-1    INDLHTELET....TP.....PPSSSALT.........TYPVKEE   333
BcaICE2-2    INDLHTELES....T......PSSSSGLTP...TPQTLPHRVKEE   335
BoICE2-1     INDLHTELES....T......PSSSSGLTP...SPQTLPYRVKEE   336
BoICE2-2     INDLHTELES....TA.....PPSSSSLNPLTPTTQTLSYRVKEE   344
BnICE2-1     INDLHTELES....T......PSSSSGLTP...SPQTLPYRVKEE   366
BnICE2-2     INDLHTELES....TA.....PPSSSSLNPLTPTTQTLSYRVKEE   333
BnICE2-3     INDLHTELES....T......PSSSSGLTP...SPQTLPYRVKEE   336
BnICE2-4     INDLHTELES....TA.....PPSSSSLNPLTPTTQTLSYRVKEE   344
BniICE2-1    INDLHTELET....TP.....PPSSSALT.........TYPVKEE   341
BniICE2-2    INDLHTELES....T......PSSSSGLTP...TPQTLPHRVKEE   335
BjuICE2-1    INDLHTELES....T......PSSSSGLTP...SPQTLPYRVKEE   338
BjuICE2-2    INDLHTELES....TA.....PPSSSSLNPLTPTTQKLSYRVKEE   332
BjuICE2-3    INDLHTELET....TP.....PPSSSALT.........TYPVKEE   341
BjuICE2-4    INDLHTELES....T......PSSSSGLTP...SPQTLPHRVKEE   335
Consensus    in lh el             p                   vkee
```

```
AtICE1        LCPSSL..PSPKGQQARVEVRLREGRAVNIHMFCGRRPGLLLATM   438
BrICE1-1      LCPSSL..PSPKGQQARVEVRLREGRAVNIHMFCGRRPGLLLATM   424
BrICE1-2      LCPSSL..PSPKGQQARVEVRLREGRAVNIHMFCGRRPGLLLATM   445
BraICE1-1     LCPSSL..PSPKGQQARVEVRLREGRAVNIHMFCGGRPGLLLATM   386
BraICE1-2     LCPSSL..PSPKGQQARVEVRVREGRAVKIHMFCGRRPGLLLATM   362
BcaICE1-1     LCPSSL..PSPKGQQARVEVRLREGRAVNIHMFCGRRPGLLLATM   393
BcaICE1-2     LCPSSL..PSPKGQQARVEVRLREGRAVNIHMFCGRRPGLLLDTM   426
BcaICE1-3     LCPSSL..PSPKGQQARVEVRLREGRAVNIHMFCGRRPGLLLATM   471
BoICE1-1      LCPSSL..PSPKGQQARVEVRLREGRAVNIHMFCGRRPGLLLDTM   399
BoICE1-2      LCPSSL..PSPKGQQARVEVRLREGRAVNIHMFCGRRPGLLLATM   445
BnICE1-1      LCPSSL..PSPKGQQARVEVRLREGRAVNIHMFCGRRPGLLLATM   423
BnICE1-2      LCPSSL..PSPKGQQARVEVRLREGRAVNIHMFCGRRPGLLLATM   441
BnICE1-3      LCPSSL..PSPKGQQARVEVRVREGRAVNIHMFCGRRPGLLLATM   335
BnICE1-4      LCPSSL..PSPKGQQARVEVRLREGRAVNIHMFCGRRPGLLLDTM   383
BnICE1-5      LCPSSL..PSPKGQQARVEVRLREGRAVNIHMFCGRRPGLLLATM   443
BnICE1-6      LCPSSL..PSSKGQQARVEVRVREGRAVNIHMFCGRRPGLLLATM   346
BniICE1-1     LCPSSL..PSPKGQQARVEVRLREGRAVNIHMFCGRRPGLLLATM   390
BniICE1-2     LCPSSL..PSPKGQQARVEVRLREGRAVNIHMFCGRRPGLLLATM   447
BniICE1-3     .CPSSL..PSPKGQQARVEVRLREGRAVNIHMFCGRRPGLLLTTM   403
BjuICE1-1     LCPSSL..PSPKGQQARVEVRLREGRAVNIHMFCGGRPGLLLATM   383
BjuICE1-2     LCPSSL..PSPKGQQARVEVRLREGRAVNIHMFCGRRPGLLLATM   442
BjuICE1-3     LCPSSL..PSPKGQQARVEVRVREGRAVNIHMFCGRRPGLLLATM   377
BjuICE1-4     LCPSSL..PSPKGQQARVEVRLREGRAVNIHMFCGRRPGLLLATM   444
BjuICE1-5     LCPSSL..PSPKGQQARVEVRLREGRAVNIHMFCGRRPGLLLATM   393
BjuICE1-6     .CPSSL..PSPKGQQARVEVRLREGRAVNIHMFCGRRPGLLLTTM   403
AtICE2        LCPSSS.LPSPKGQQPRVEVRLREGKAVNIHMFCGRRPGLLLSTM   394
BrICE2-1      LCPSSSSLPSPKGEQARIEVKLREGKAVNIHMFCGRRPGLLLSTM   385
BrICE2-2      LCPSSS.FPSPRGEQARIEVKLREGKAVNIHMFCGRRPGLLLSTM   374
BraICE2-1     LCPSSL..PSPKGQQARVEVRLREGRAVNIHMFCGRRPGLLLATM   328
BraICE2-2     LCPSSS.FPSPRGEQARIEVKLREGKAVNIHMFCGRRPGLLLSTM   377
BcaICE2-1     LCLSSS.LPSPRGQEARIEVKLREGKAVNIHMFCGRRPGLLLSTM   377
BcaICE2-2     LCPSSS.LPSPKGQQARIEVNLREGKAVNIHMFCGRRPGLLLSTM   379
BoICE2-1      LCPSSSSLSSPKGEQARIEVKLREGKAVNIHMFCGRRPGLLLSTM   381
BoICE2-2      LCPSSS.FPSPRGEQARIEVKLREGKAVNIHMFCGRRPGLLLSTM   388
BnICE2-1      LCPSSSSLPSPKGEQARIEVKLREGKAVNIHMFCGRRPGLLLSTM   411
BnICE2-2      LCPSSS.FPSPRGEQARIEVKLREGKAVNIHMFCGRRPGLLLSTM   377
BnICE2-3      LCPSSSSLSSPKGEQARIEVKLREGKAVNIHMFCGRRPGLLLSTM   381
BnICE2-4      LCPSSS.FPSPRGEQARIEVKLREGKAVNIHMFCGRRPGLLLSTM   388
BniICE2-1     LCLSSS.LPSPRGQEARIEVKLREGKAVNIHMFCGRRPGLLLSTM   385
BniICE2-2     LCPSSS.LPSPKGQQARIEVNLREGKAVNIHMFCGRRPGLLLSTM   379
BjuICE2-1     LCPSSSSLPSPKGEQARIEVKLREGKAVNIHMFCGRRPGLLLSTM   383
BjuICE2-2     LCPSSS.FPSPRGEQARIEVKLREGKAVNIHMFCGRRPGLLLSTM   376
BjuICE2-3     LCLSSS.LPSPRGQEARIEVKLREGKAVNIHMFCGRRPGLLLSTM   385
BjuICE2-4     LCPSSS.LPSPKGQQARIEVNLREGKAVNIHMFCGRRPGLLLSTM   379
Consensus      c ss    s  g   r ev  reg av ihmfcg rpglll tm
```

```
AtICE1      KALDNLGLDVQQAVISCFNGFALDVFRAEQCQEGQEILPDQIKAV   483
BrICE1-1    KALDNLGLDVQQAVISCFNGFALDVFRAEQCQEGQEILPDQIKAV   469
BrICE1-2    KALDNLGLDVQQAVISCFNGFALDVFRAEQCQEGQEILPDQIKAV   490
BraICE1-1   KALDNLGLDVQQAVISCLNGFALDVFRAEQCQEGQEILPDQIKAV   431
BraICE1-2   KALDNLGLDVQQAVISCFNGFALDVFRAEQCQEGQEILPDQIKAV   407
BcaICE1-1   KALDNLGLDVQQAVISCFNGFALDVFRAEQCQEGQEILPDQIKAV   438
BcaICE1-2   KALDNLGLDVQQAVISCFNGFALDVFRAEQCQEGQEILPDQIKAV   471
BcaICE1-3   KALDNLGLDVQQAVISCFNGFALDVFRAEQCQEGQEILPDQIKAV   516
BoICE1-1    KALDNLGLDVQQAVISCFNGFALDVFRAEQCQEGQEILPDQIKAV   444
BoICE1-2    KALDNLGLDVQQAVISCFNGFALDVFRAEQCQEGQEILPDQIKAV   490
BnICE1-1    KALDNLGLDVQQAVISCFNGFALDVFRAEQCQEGQEILPDQIKAV   468
BnICE1-2    KALDNLGLDVQQAVISCFNGFALDVFRAE............VRTI   474
BnICE1-3    KALDNLGLDVQQAVISCFNGFALDVFRAEQCQEGQEILPDQIKAV   380
BnICE1-4    KALDNLGLDVQQAVISCFNGFALDVFRAEQCQEGQEILPDQIKAV   428
BnICE1-5    KALDNLGLDVQQAVISCFNGFALDVFRAEQCQEGQEILPDQIKAV   488
BnICE1-6    KALDNLGLDVQQAVISCFNGFALDVFRAEKCQEGHEILPDQIKAV   391
BniICE1-1   KALDNLGLDVQQAVISCFNGFALDVFRAEQCQEGQEILPDQIKAA   435
BniICE1-2   KALDNLGLDVQQAVISCFNGFALDVFRAEQCQEGQEILPDQIKAV   492
BniICE1-3   KALDNLGLDVQQAVISCFNGFALDVFRAEQCQEGHDILPDQIKAL   448
BjuICE1-1   KALDNLGLDVQQAVISCLNGFALDVFRAEQCQEGQEILPDQIKAV   428
BjuICE1-2   KALDNLGLDVQQAVISCFNGFALDVFRAEQCQEGQEILPDQIKAV   487
BjuICE1-3   KALDNLGLDVQQAVISCFNGFALDVFRAEQCQEGQEILPDQIKAV   422
BjuICE1-4   KALDNLGLDVQQAVISCFNGFALDVFRAEQCQEGQEILPDQIKAV   489
BjuICE1-5   KALDNLGLDVQQAVISCFNGFALDVFRAEQCQEGQEILPDQIKAV   438
BjuICE1-6   KALDNLGLDVQQAVISCFNGFALDVFRAEQCQEGHDILPDQIKEL   448
AtICE2      RALDNLGLDVQQAVISCFNGFALDVFRAEQCQEDHDVLPEQIKAV   439
BrICE2-1    RALDDLGLDVQQAVISCFNGFALDVFRAEQCQEGHEVFPEQIKAV   430
BrICE2-2    RALDNLGLDVQQAVVSCFNGFALDVFRAEQCQEGHDVVPEQIKAV   419
BraICE2-1   KALDNLGLDVQQAVISCFNGFALDVFRAEQCQEGQEILPDQIKAV   373
BraICE2-2   RALDNLGLDVQQAVVSCFNGFALDVFRAEQCQEGHDVVPEQIKAV   422
BcaICE2-1   RALDNLGLDVQQAVVSCFNGFALDVFRAEQCQEGHDVVPEQIKAV   422
BcaICE2-2   RALDDLGLDVQQAVISCFNGFALDVFRAEQCQEGQEVLAEQIKAV   424
BoICE2-1    RALDDLGLDVQQAVISCFNGFALDVFRAEQCQEGHEVFPEQIKAV   426
BoICE2-2    RALDNLGLDVQQAVVSCFNGFALDVFRAEQCQEGHDVVPEQIKAV   433
BnICE2-1    RALDDLGLDVQQAVISCFNGFALDVFRAEQCQEGHEVFPEQIKAV   456
BnICE2-2    RALDNLGLDVQQAVVSCFNGFALDVFRAEQCQEGHDVVPEQIKAV   422
BnICE2-3    RALDDLGLDVQQAVISCFNGFALDVFRAEQCQEGHEVFPEQIKAV   426
BnICE2-4    RALDNLGLDVQQAVVSCFNGFALDVFRAEQCQEGHDVVPEQIKAV   433
BniICE2-1   RALDNLGLDVQQAVVSCFNGFALDVFRAEQCQEGHDVVPEQIKAV   430
BniICE2-2   RALDDLGLDVQQAVISCFNGFALDVFRAEQCQEGQEVLAEQIKAV   424
BjuICE2-1   RALDDLGLDVQQAVISCFNGFALDVFRAEQCQEGHEVFPEQIKAV   428
BjuICE2-2   RALDNLGLDVQQAVVSCFNGFALDVFRAEQCQEGHDVVPEQIKAV   421
BjuICE2-3   RALDNLGLDVQQAVVSCFNGFALDVFRAEQCQEGHDVVPEQIKAV   430
BjuICE2-4   RALDDLGLDVQQAVISCFNGFALDVFRAEQCQEGQEVLAEQIKAV   424
Consensus    ald lgldvqqav sc ngfaldvfrae
```

AtICE1	LFDTAGYAGMI	494
BrICE1-1	LLDTAGYAGMI	480
BrICE1-2	LFDTAGYAGMI	501
BraICE1-1	LFDTAGYAGMI	442
BraICE1-2	LLDTAGYAGLI	418
BcaICE1-1	LFDTAGYAGMI	449
BcaICE1-2	LFDTAGYAGMI	482
BcaICE1-3	LFDTA..ATLV	525
BoICE1-1	LFDTAGYAGMI	455
BoICE1-2	LFDTAGYAGMI	501
BnICE1-1	LLDTAGYAGMI	479
BnICE1-2	TIHVP.FTSNI	484
BnICE1-3	LLDTAGYAGLI	391
BnICE1-4	LFDTAGYAGMI	439
BnICE1-5	LFDTAGYAGMI	499
BnICE1-6	LLDTASDEVEC	402
BniICE1-1	LFDTAGYAGMI	446
BniICE1-2	LFDTAGYAGMI	503
BniICE1-3	LFDIAGYPDMI	459
BjuICE1-1	LFDTAGYAGMI	439
BjuICE1-2	LFDTAGYAGMI	498
BjuICE1-3	LLDTAGYAGLI	433
BjuICE1-4	LFDTAGYAGMI	500
BjuICE1-5	LFDTAGYAGMI	449
BjuICE1-6	LFDIAGYPDMI	459
AtICE2	LLDTAGYAGLV	450
BrICE2-1	LLDTAGYSGLL	441
BrICE2-2	LLDTVGYTGLV	430
BraICE2-1	LFDTAGYAGMI	384
BraICE2-2	LLDTVGYTGLV	433
BcaICE2-1	LLDTVGYTGLV	433
BcaICE2-2	LLDTAGYSGLL	435
BoICE2-1	LLDTAGYSGLL	437
BoICE2-2	LLDTVGYTGLV	444
BnICE2-1	LLDTAGYSGLL	467
BnICE2-2	LLDTVGYTGLV	433
BnICE2-3	LLDTAGYSGLL	437
BnICE2-4	LLDTVGYTGLV	444
BniICE2-1	LLDTVGYTGLV	441
BniICE2-2	LLDTAGYSGLL	435
BjuICE2-1	LLDTAGYSGLL	439
BjuICE2-2	LLDTVGYTGLV	432
BjuICE2-3	LLDTVGYTGLV	441
BjuICE2-4	LLDTAGYSGLL	435
Consensus		
