# Supplementary figures and images for: Brassica rapa BrICE1 and BrICE2 Positively Regulate the Cold Tolerance via CBF and ROS Pathways, Balancing Growth and Defense in Transgenic Arabidopsis
Source: Plants (Basel). 2024 Sep 20;13(18):2625. doi: 10.3390/plants13182625 (PMC11435425; doi:10.3390/plants13182625)

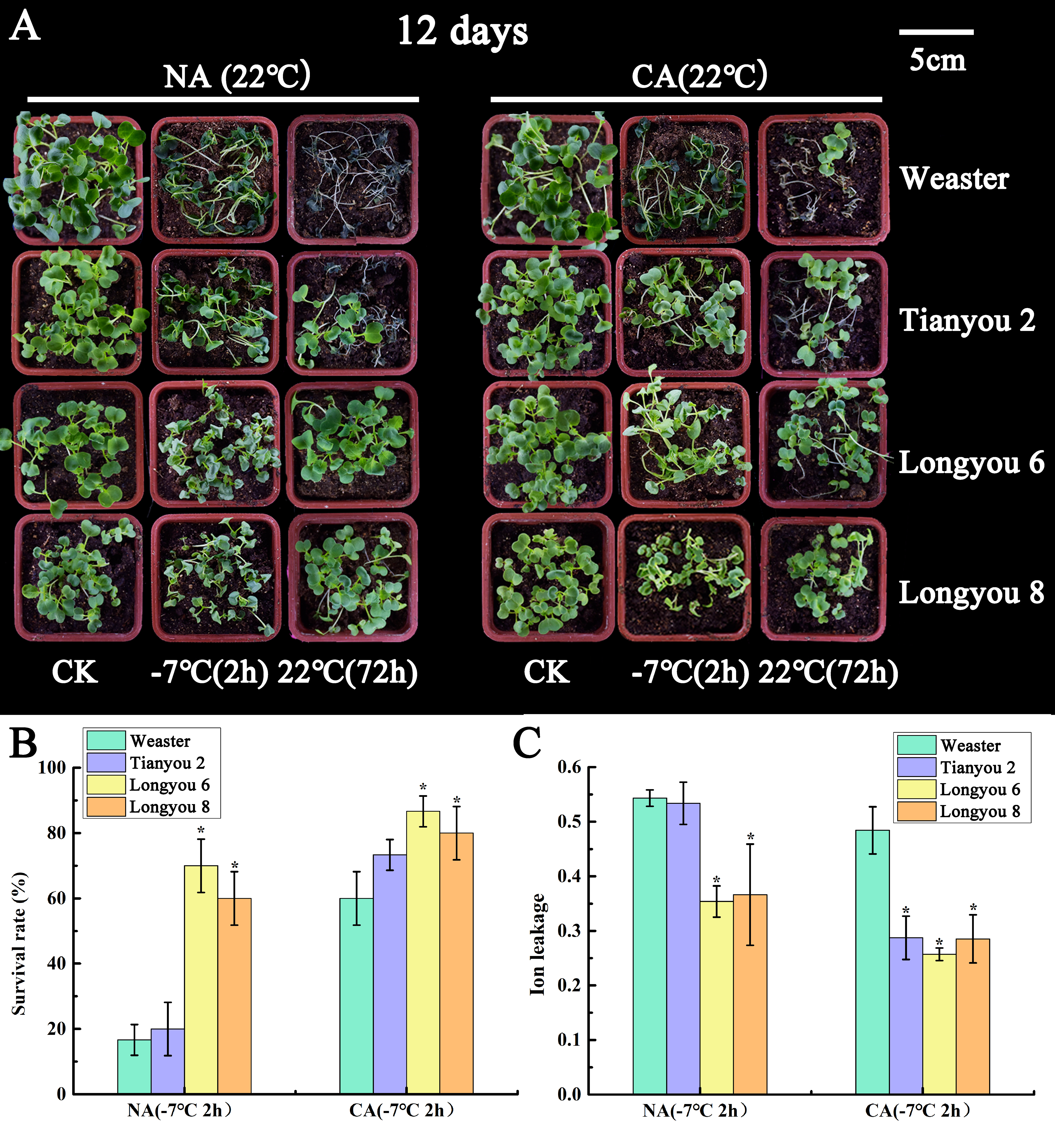

Supplement: Supplementary file 1 [file plants-13-02625-s001.zip › Supplementary Figure S2.tif]

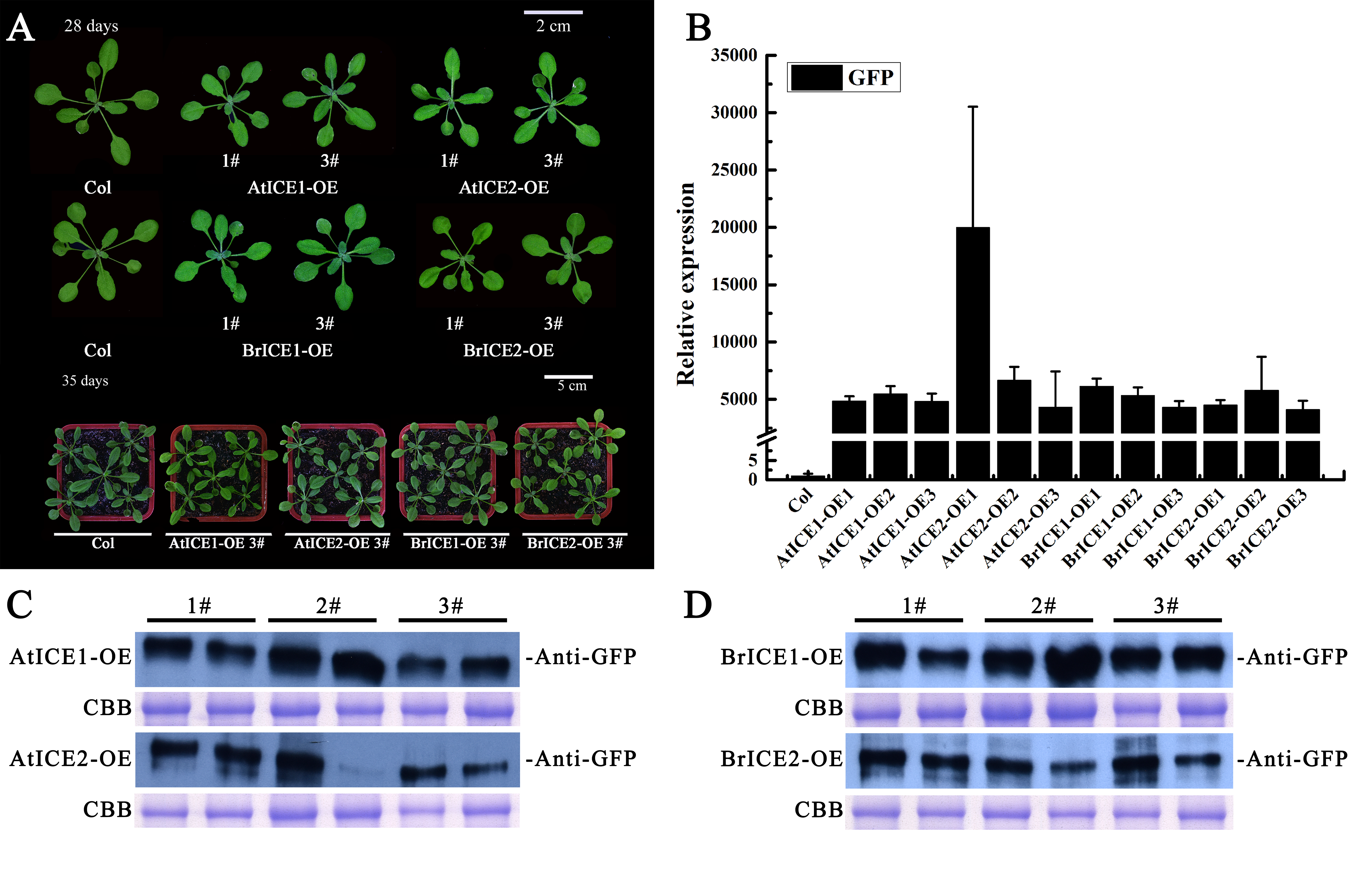

Supplement: Supplementary file 1 [file plants-13-02625-s001.zip › Supplementary Figure S3.tif]

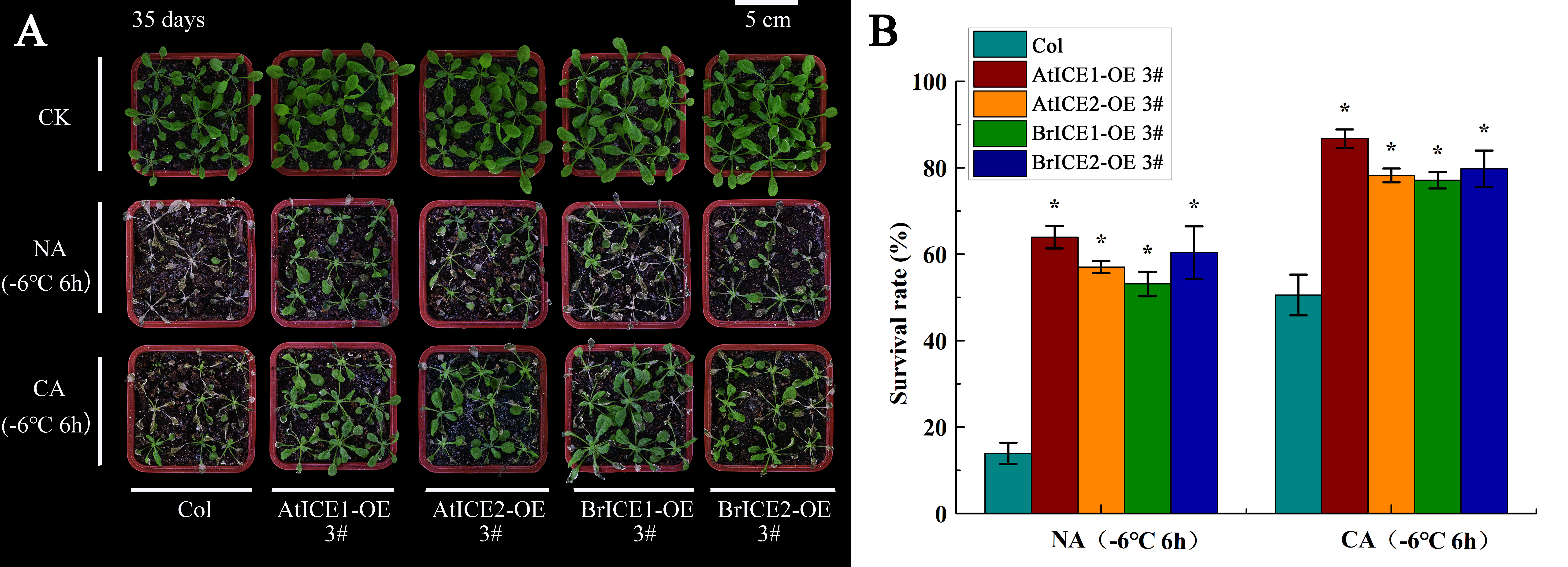

Supplement: Supplementary file 1 [file plants-13-02625-s001.zip › Supplementary Figure S4.tif]

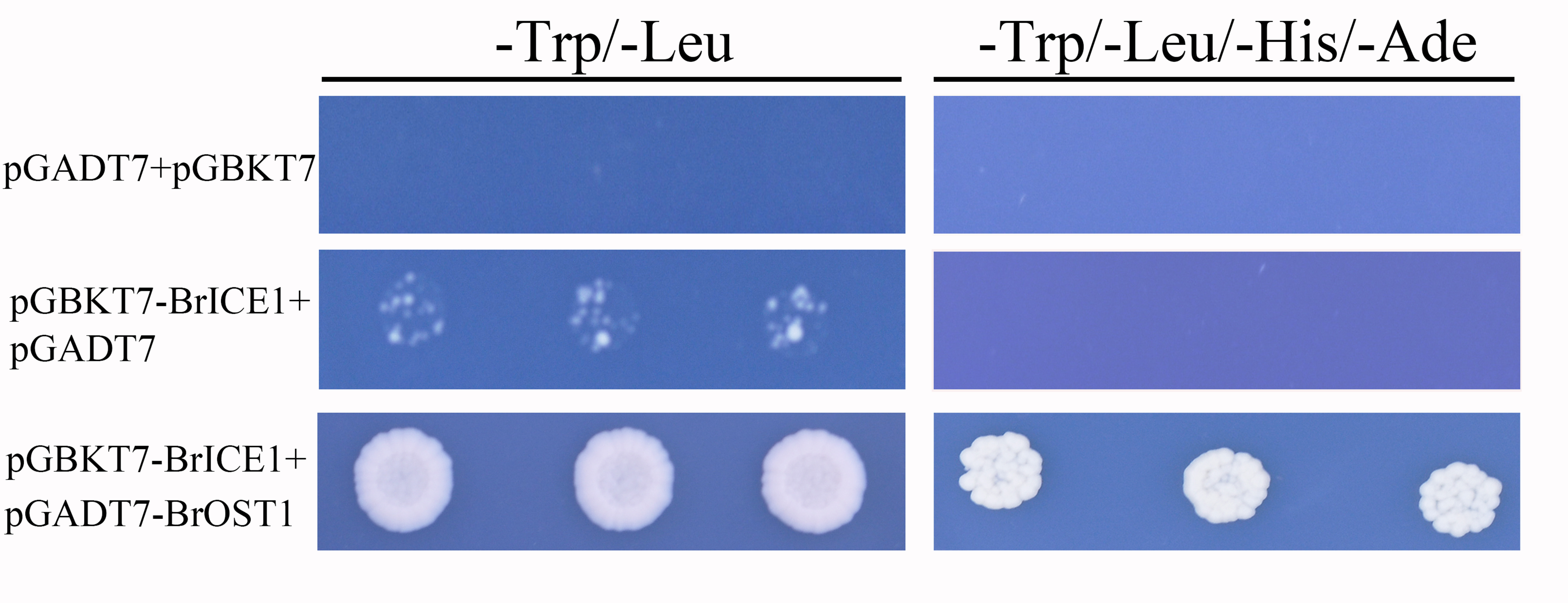

Supplement: Supplementary file 1 [file plants-13-02625-s001.zip › Supplementary Figure S5.tif]

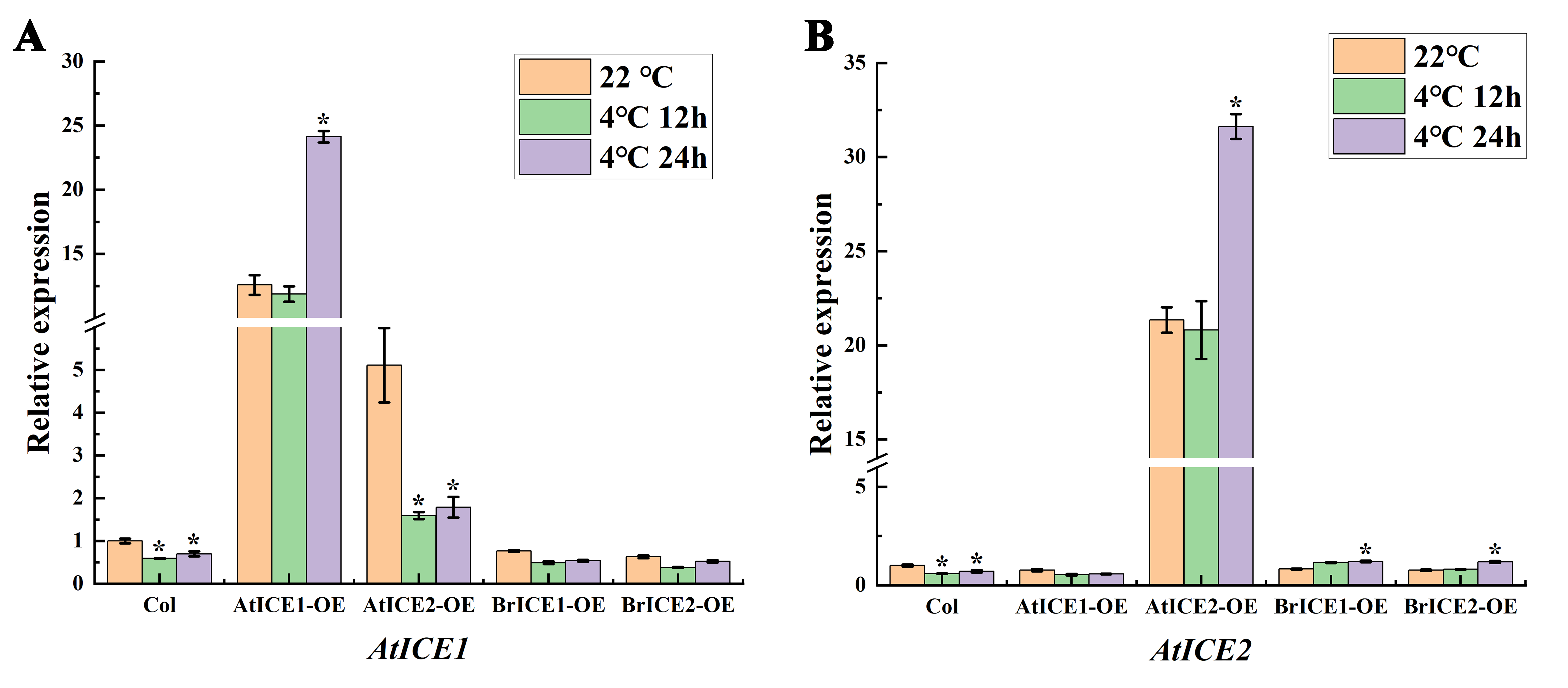

Supplement: Supplementary file 1 [file plants-13-02625-s001.zip › Supplementary Figure S6.tif]
